# Glycomic profiling of carcinoembryonic antigen isolated from human tumor tissue

**DOI:** 10.1186/s12014-015-9088-3

**Published:** 2015-06-27

**Authors:** Chuncui Huang, Tiancheng Zhan, Yaming Liu, Qianqian Li, Hongmei Wu, Dengbo Ji, Yan Li

**Affiliations:** Institute of Biophysics, Chinese Academy of Sciences, Beijing, 100101 P. R. China; Key laboratory of Carcinogenesis and Translational Research (Ministry of Education), Department of Colorectal Surgery, Peking University Cancer Hospital & Institute, No. 52 Fucheng Rd., Haidian District, Beijing, 100142 P. R. China

**Keywords:** Carcinoembryonic antigen, Glycan profiling, MALDI-TOF-MS^3^

## Abstract

**Background:**

Carcinoembryonic antigen (CEA) is a protein commonly found in human serum, with elevated CEA levels being linked to the progression of a wide range of tumors. It is currently used as a biomarker for malign tumors such as lung cancer and colorectal cancer [Urol Oncol: Semin Orig Invest 352: 644–648, 2013 and Lung Cancer 80: 45-49, 2013]. However, due to its low specificity in clinical applications, CEA can be used for monitoring only, rather than tumor diagnosis. The function of many glycoproteins is critically dependent on their glycosylation pattern, which in turn has the potential to serve in tumor detection. However, little is known about the detailed glycan patterns of CEA.

**Methods:**

To determine these patterns, we isolated and purified CEA proteins from human tumor tissues using immunoaffinity chromatography. The glycan patterns of CEA were then analyzed using a Matrix-Assisted Laser Desorption/Ionization-Time of Flight-Mass Spectrometry^3^ (MALDI-TOF-MS^3^) approach.

**Results:**

We identified 61 glycoforms in tumor tissue, where CEA is upregulated. These glycosylation entities were identified as bi-antennary, tri-antennary and tetra-antennary structures carrying sialic acid and fucose residues, and include a multitude of glycans previously not reported for CEA.

**Conclusion:**

Our findings should facilitate a more precise tumor prediction than currently possible, ultimately resulting in improved tumor diagnosis and treatment.

**Electronic supplementary material:**

The online version of this article (doi:10.1186/s12014-015-9088-3) contains supplementary material, which is available to authorized users.

## Background

Human Carcinoembryonic Antigen (CEA) is a glycoprotein that is expressed during embryonal development [1]. It was identified in human cancer tissues, and is now known to be related to the progression of tumors [2, 3]. As a tumor marker, CEA has been utilized in the monitoring of tumor, judgment of neoplasm staging, as well as prediction of tumor recurrence [4]. However, further investigation revealed that detection of this antigen alone is not sufficiently specific for tumor diagnostics. Therefore, improvement in specificity of CEA detection remains a challenge for clinical tumor diagnosis [5].

The proper functioning of glycoproteins is directly related to the nature of their carbohydrate moiety changes in protein, and dysregulation of glycosylation patterns appears to play a crucial role in the pathogenesis and progression of various diseases. It was reported that an increase in fucosylation and formation of new glycan antennae are characteristic features of carbohydrate chains that appears to associate with the occurrence of related diseases [6]. For example, core fucosylation (CF)-glycosylation of an α-fetoprotein isoform (AFP-L3) was approved as a biomarker of hepatocellular carcinoma (HCC) by US Food and Drug Administration (FDA) [7]. Similar to AFP-L3, the use of CEA glycoprotein pattern has the potential to improve the specificity of tumor diagnosis.

CEA contains 28 potential N-liked glycosylation sites, and the glycofraction includes mannose, galactose, N-acetylglucosamine, fucose and sialic acid [8]. The glycan composition of CEA displays a considerable heterogeneity in the sugar content of the antenna, but detailed structures of these glycans have so far not been reported [9]. One earlier study identified the core structures of CEA oligosaccharides and proposed that CEA contained approximate 40 N-linked oligosaccharide chains [10]. In a recent study, approximate 25 N-linked carbohydrate structures of CEA were determined, and complex-type and high mannose-type carbohydrate chains were analyzed [9].

Various techniques are currently in use to analyze glycans, including liquid chromatography (LC), lectin microarray, exoglycosidases digestion, capillary electrophoresis (CE) and MALDI-TOF-MS^n^. Methods involving LC and exoglycosidase digestion require large amounts of sample material, and are therefore not suitable for clinical application. In addition, specific glycan structures cannot be obtained by lectin microarray analysis to investigate carbohydrate moieties. Moreover, standard and purified glycans are required for CE analysis. Except for MALDI-TOF-MS^n^, all of the methods for structure analysis of CEA glycans mentioned above are sample/standard-consuming as well as cost-intensive. More importantly, specific glycan structures of CEA have not been entirely resolved. In contrast, MALDI-TOF-MS^n^ is both sensitive and can be used in high through-put mode. Furthermore, uncertain fragments of glycans can be further decomposed until specific and detailed structures are obtained. As a result, components, sequence and branches of glycans can be specifically obtained by using MALDI-TOF-MS^n^. In order to investigate glycan structures of humanized CEA, we first extracted CEA from colon tumor using monoclonal anti-CEA coupled to activated Sepharose 4B. Glycan structures of extracted CEA were then analyzed using MALDI-TOF-MS^3^, which allowed for sensitive identification of a multitude of previously unknown glycan structures.

## Methods

### Materials

Trihydroxymethylaminomethane, sodium chloride, sodiumdeoxycholate, sodium dodecyl sulfate, disodium hydrogen phosphate, sodium phosphate monobasic sodium carbonate, hydrochloric acid, ammonium bicarbonate, sodium hydroxide, standard peptides, glycine, ethanolamine, propanol, DL-dithiothreitol (DTT), iodoacetamide (IAA), cyanogens bromide, acetic acid and phosphate buffer were purchased from Sigma-Aldrich. Acetonitrile, ethanol and water were of analytical grade and obtained from Avantor Performance Materials, Inc. (PA, USA). Mouse anti-CEA antibody was purchased from ZSG-BIO (Beijing, China). Trypsin and peptide N-glycosidase F (PNGase F) were purchased from Promega Corporation (WI, USA). C_18_-Sep-Pak cartridges were obtained from Waters Corporation (MA, USA). Clinical samples of rectum loops from patients with colorectal carcinoma were provided by Beijing Cancer Hospital.

### CEA extraction and purification

Colorectal carcinoma tissue was placed on ice, and blood was washed off using 0.01 M PBS buffer. Adiposetissue was cut into pieces and RIPA lysis buffer (0.15 g Tris, 0.438 g NaCl, 0.05 g NaOH, 0.5 g sodiumdeoxycholate, and 0.05 g SDS) was added to the tissue pieces. The mixture was grinded into homogenate in a tissue grinder. All the tissue homogenate was ultrasonically decomposed thoroughly in the lysis buffer. Then the tissue homogenate was centrifuged at 4000 g, and the supernatant was kept on ice, at −80 **°**C.

**Fig. 1:**
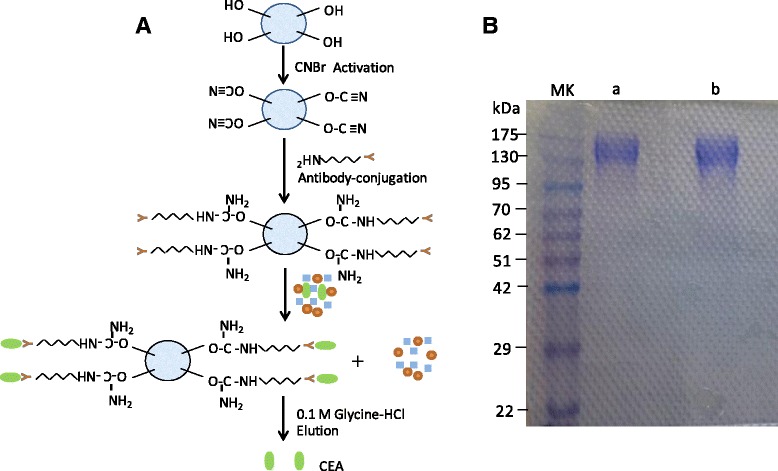
Scheme of CEA extraction and purification (**a**), and SDS-PAGE analysis of CEA and molecular weight markers (**b**). Lanes of MW, a and b represent molecular weight markers, CEA standard and extracted CEA from colorectal carcinoma patients respectively

Sepharose 4B stored in 20 % ethanol was applied onto a cartridge, and activated with cyanogens bromide solution in the presence of 2 M Na_2_CO_3_. Mouse anti-CEA was mixed and conjugated with the activated gel on the cartridge overnight at 4 **°**C. Remaining activated groups were blocked with 0.2 M ethanolamine (pH 7 ~ 8), and any excess of uncoupled proteins were washed off by PBS buffer (pH = 7.4) for three times. 0.5 mL of supernatant of the homogenate was fractionated overnight at 4 **°**C using an anti-CEA immunoadsorbent cartridge. Target CEA was eluted from the cartridge using 0.1 M glycine-HCl (pH 2.4) after washing with PBS buffer three times. The purity of eluted CEA was confirmed using sodium dodecyl sulfate polyacrylamide gel electrophoresis (SDS-PAGE, see Fig. 1b). Afterwards, the concentration of eluted CEA was determined using an enzyme-linked immunoadsorbent assay (ELISA).

### N-glycan release

Purified CEA was first reduced and carboxymethylated in the presence of DTT and IAA followed by dialysis in 50 mM Ambic buffer for 24–48 h at 4 **°**C. Glycoproteins were further incubated at 37 °C overnight to be cleaved into glycopeptides by tryptic digestion. First, the Sep-Pak® C_18_ cartridge was conditioned with methanol, 5 % acetic acid and the 100 % propan-1-ol, and then glycopeptide samples were applied to the cartridge. Second, samples were eluted stepwise with 20 % propan-1-ol solution and 40 % propan-1-ol solution. Third, the propanol Sep-Pak fractions were digested at 37 °C for 20–24 h using PNGase F. Separation of the N-glycans from the mixtures was conducted using a Sep-Pak® C_18_ propanol/5 % acetic acid system. Finally, permethylation and subsequent purification were performed according to previously described [11].

### Structure analysis of N-linked glycans released from extracted CEA

Fractions of N-linked glycan mixtures from extracted CEA were analyzed using an Axima MALDI Resonance mass spectrometer (Shimadzu). A nitrogen laser was used to irradiate samples at 337 nm, and an average of 200 shots was taken. An aqueous solution of N-linked oligosaccharide mixture (ca. 100 pmol, 0.5 μL) was applied to a *μ*focus MALDI plate target (900 μm, 384 circles, HST Inc.). A solution (0.5 μL) of 2,5-dihydroxybenzoic acid (DHB, 20 mg/mL) in a mixture of methanol–water (1:1) containing 0.1 % trifluoroacetic acid (TFA) and 1 mM Na^+^ were added to the plate, and then mixed with oligosaccharides. Finally, the mixture was exposed to air at room temperature until dry. During MS, the ion peaks were calibrated using a mixture of standard peptides as mass markers.

## Results

### CEA level extracted

**Fig. 2:**
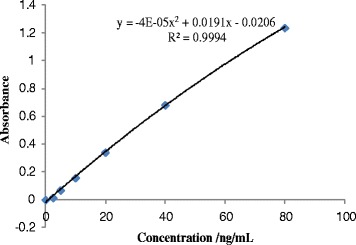
Calibration curve of standard CEA using ELISA

In order to analyze the glycan moieties of CEA, we first isolated and purified CEA protein by immunoadsorption from human tumor tissues. CEA antibodies were first covalently coupled to Sepharose beads, to then allow target CEA to be precipitated, before it was eluted from the cartridge (Fig. 1a). Non-specific binding, resulting in low signal-to-noise-ratio, was avoided through covalent coupling of CEA antibodies. The concentration of eluted CEA was determined using an ELISA method. Using a standard curve (Fig. 2), we estimated that our procedure yielded approximate 12 ng CEA glycoproteins per 400 mg of colorectal carcinoma tissue used.

### Analysis of N-linked glycan structures of extracted CEA

To investigate the specific glycan structures present in CEA, we analyzed our samples using MALDI-TOF-MS^3^. We detected the presence of 61 unique glycans in the extracted CEA. These glycans were detected without further separation, and were found to be all linked to asparagine (see Fig. 3 and Additional file 1: Table S1). In addition, the proposed N-glycan assignments for all peaks are shown. First, we analyzed the terminal structures of glycans of CEA. Furthermore, MS/MS/MS analysis of the assigned glycans was performed (see Additional file 2: Figures S1-S61). The spectra showed that of the 61 glycan moieties, 6 N-linked glycans were of high mannose-type, 23 were of bi-antennary structure, 14 were of a tri-antennary structure, and 17 were of tetra-antennary structure. Interestingly, one glycan with hybrid structure was determined as well.

**Fig. 3:**
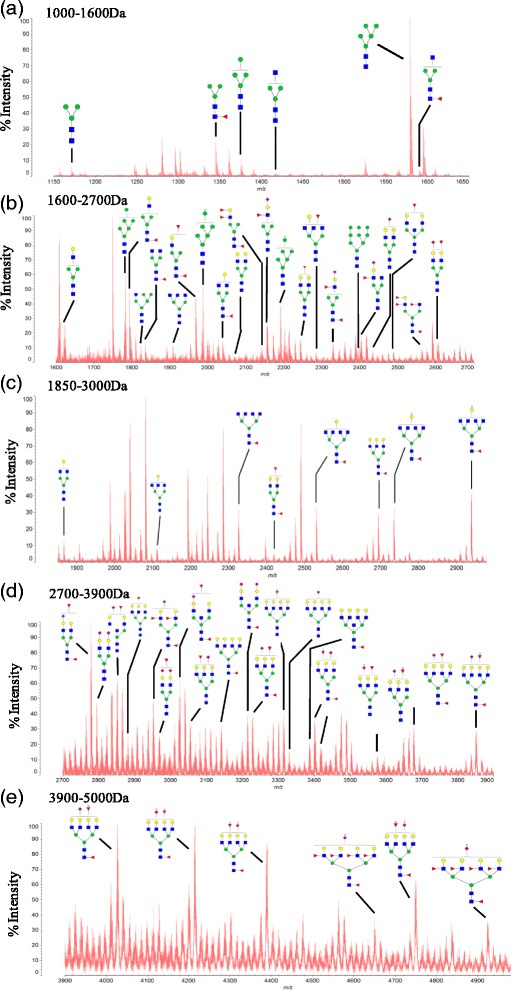
MALDI-TOF MS profiles of the permethylated N-linked glycans from CEA of colorectal carcinoma patients. All molecular ions are present in singly charged sodiated form ([M + Na]^+^). In the glycan structure, blue square refers to *N*-acetylglucosamine (■), green circle refers to mannose (●), yellow circle refers to Galactose (●), red triangle refers to Fucose (▲), and purple diamond refers to N –acetylneuraminic acid (♦). a-e refers to mass spectrum of CEA glycans in different mass range

As expected, more than half of the 61 N-linked glycans contained one or two residues of sialic acid attached to the terminal galactosyl group. Intriguingly, more than half of these glycans contained as many as two residues of fucose. These fucose molecules were linked either to a reducing terminal N-acetylglucosaminide, or a non-reducing end of the glycans. In addition, 23 N-linked glycans were found to contain both sialic acid and fucose residues. Interestingly, 23 N-linked glycans contained a SialylLewis^x^ terminal structure, thus accounting for approximate 38 % of all glycan moieties determined. The presence and determined level of SialylLewis^x^ structure are in agreement with those reported in earlier studies, where SialylLewis^x^ was identified as a cancer marker [12].

Second, we investigated the core structures of the CEA glycan moeties. 31 CEA glycans (approximate 50 %) contained core fucosylation. During core fucosylation, fucose is transferred from guanosinediphosphate-fucose (GDP-Fuc) to the innermost GlcNAc catalyzed by core fucosyltransferase [7]. Similar to SialylLewis^x^, these results are in agreement with the literature describing core fucosylation as a cancer marker [13, 14]. In conclusion, our method is sensitive, and can be used for glycan detection of clinical samples. Our analysis indicated that tumor CEA proteins contain high levels of glycan structures such as SialylLewis^x^ and core fucosylation, which have previously been associated with tumor tissue.

## Discussion and conclusions

We identified 61 different glycosylated forms of CEA in the tumor samples analyzed. These structures included bi-antennary, tri-antennary and tetra-antennary structures carrying sialic acid and fucose residues. In addition, we showed that the presence of SialylLewis^x^ and core fucosylation structures could facilitate the specificity improvement of CEA for cancer detection.

### Glycan structures

Glycans possess several important features, including the ability of different types and numbers of sugar residues to form glycosidic bonds with one another, the type of anomeric linkage, the position and the absence or presence of branching. Together, these characteristics give rise to the complex heterogeneity in living systems. If some of these characteristics change, transformation of cells or tissues may occur. Therefore, glycan structure analysis should greatly improve the specificity and sensitivity of potential biomarkers that at present are not sufficiently specific for clinical tumor diagnosis. Currently, detection of CEA is not specific enough for adequate diagnosis of colonic neoplasia, and therefore CEA levels are only monitored as a prognostic indicator therapy response. Investigation of CEA glycans structures such as SialylLewis^x^ units and fucosylation levels will greatly contribute to a more specific diagnosis of colorectal carcinoma.

The determined SialylLewis^x^ structure here is in agreement with previous studies that reported the presence of SialylLewis^x^ in malignancies [15–22]. SialylLewis^x^ is an adhesion molecule present on O-and N-linked oligosaccharides, and is therefore of great significance in cell-cell recognition [23]. Importantly, SialylLewis^x^ is thought to be associated with metastatic ability of tumor cells, and has the potential to be used as a marker for pathological tumor extensiveness, e.g., nodal extension or distant metastasis [24]. It was reported earlier that SialylLewis^x^ is a tumor-specific glycan marker used for tumor staging, prognosis and progression, and was found to be associated with proteins such as alpha 1-acid glycoprotein, CD66, and MUC7 [25–28]. It has also been demonstrated that SialylLewis^x^ is present at elevated levels in primary breast carcinoma [29]. Fucosylation levels increased significantly when malignant transformation occurred in the tissue cells. It has been proved that core-fucosylation is involved in the regulation of many biological processes in mammals. Abnormal core-fucosylation was previously reported to be involved in human pathological processes, such as metastasis [7]. However, few reports exist that tested the use of SialylLewis^x^ and core-fucosylation as tumor markers for colorectal carcinoma. Based on our results here, the SialylLewis^x^ and core-fucosylation structures do present in CEA protein extracted from colon carcinomas tissues. The results of our present study indicate that determined levels of SialylLewis^x^ and core fucosylation of CEA proteins could be useful and specific indicators for colorectal carcinoma.

### CEA samples from human

CEA samples from human rather than recombinant proteins are essentially required to improve the diagnostic specificity of CEA for cancer detection in clinical application. Biological synthesis of glycan chains is a non-template and complicated process, and this metabolic pathway cannot be controlled as accurately as nucleic acid and proteins. Moreover, synthesis and rupture of glycosidic bonds are controlled by either glycosyltransferase or hydrolase in endoplasmic reticulum and Golgi apparatus, and these processes are extraordinarily non-deterministic. Glycan structures in recombinant proteins may be different from glycan structures synthesized via the mentioned process in the endoplasmic reticulum and Golgi apparatus of glycoproteins extracted from human patients. Consequently, CEA samples from human patients are necessary for studies about clinical application. In the present study, CEA samples were extracted and purified from patients, thus the harvested glycan structures represent exactly those synthesized in biological systems. Therefore, the determined glycan structures should assist greatly in improving the specificity of cancer diagnosis in clinical application.

### Analysis method

CEA concentrations in human samples are in the level of ng/mL, and it is therefore necessary to use highly sensitive detection methods for the analysis of its glycosylation patterns. MALDI-TOF-MS method possesses a much higher sensitivity compared to those reported previously for CEA glycan analysis. In a previous study that used limited exoglycosidase digestion and methylation analysis, the amount of CEA proteins required was almost 10 mg for glycan analysis [8]. In the present study, only 5 μg of CEA proteins were required for one spot on the MALDI plate, indicating that our method is sensitive, with the amount of material required being three magnitudes lower. In addition, permethylation of released glycans were optimized in this study, and the experiment time was shortened without effect on results. All the structures of N-linked glycans were obtained and analyzed using MALDI-TOF-MS^3^ in about 60 min after glycans were released from CEA proteins. The procedure of glycans release in this study was mainly composed of tryptic digestion, N-Glycosidase F digestion and the necessary clean-up of products by Sep-Pak purification steps respectively. However, the reported routine procedures of glycans release and structure analysis of glycan moieties of CEA proteins often include multistep enzyme digestion, liberation of glycans and complicated methylation analysis [9, 10]. Therefore, short analysis time and simple treatment process contributed to high through-put of current methods, especially when multiple clinical samples were required to be analyzed rapidly. The limitation of our method presented here is that linkage information of glycans could not be obtained, apart from branch and sequence information. Nevertheless, we believe that information about composition, branch architecture and sequences of the CEA glycan moieties should be sufficient to improve the specificity of CEA to allow its use as a colon tumor marker.

## Conclusions

A method including extraction, purification of glycoprotein CEA from tumor tissue, and characterization of CEA glycans structures was developed. Sixty-one unique oligosaccharides were obtained, and structures of these glycans were investigated. The presented methodology should greatly assist further study of CEA proteins and its application as a specific colon tumor marker, and glycans signatures may become useful markers for the distinction of cancers.

## Additional files

Additional file 1: Table S1. N-glycans harvested from CEA of colorectal carcinoma patients. Additional file 2:
**Supporting Information (Figures S1-S61).**

## Abbreviations

CEA: Carcinoembryonic antigen
MALDI-TOF-MS^n^: Matrix-Assisted Laser Desorption/Ionization-Time of Flight-Mass Spectrometry^n^
CF: Core fucosylation
HCC: Hepatocellular carcinoma
FDA: US Food and Drug Administration
LC: Liquid chromatography
CE: Capillary electrophoresis
DTT: DL-Dithiothreitol
IAA: Iodoacetamide
PNGase F: Peptide N-glycosidase F
ELISA: Enzyme-linked immunoadsorbent assay
DHB: 2,5-Dihydroxybenzoic acid
TFA: Ttrifluoroacetic acid
GDP-Fuc: Guanosinediphosphate-fucose
MS/MS/MS: Tandem mass spectrometry
m/z: Mass to charge ratio
