# Supplementary material for: Glycomic profiling of carcinoembryonic antigen isolated from human tumor tissue
Source: Clin Proteomics. 2015 Jun 27;12(1):17. doi: 10.1186/s12014-015-9088-3 (PMC4495800; doi:10.1186/s12014-015-9088-3)
Supplement: Additional file 1: Table S1. — N-glycans harvested from CEA of colorectal carcinoma patients. [file 12014_2015_9088_MOESM1_ESM.pdf]

Table 1. N-glycans harvested from CEA of colorectal carcinoma patients

Peak No.	Measured N-glycan Mass [M+Na]+	Theoretical N-glycan Mass [M+Na]+	N-glycan Structure
1	1171.423	1171.583	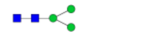
2	1345.385	1345.672	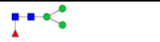
3	1375.377	1375.683	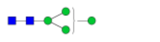
4	1416.344	1416.709	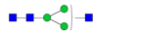
5	1579.327	1375.783	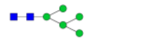
6	1620.301	1620.809	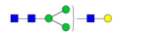
7	1783.241	1783.882	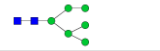
8	1794.227	1794.898	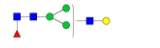
9	1866.002	1865.935	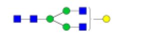
10	1824.220	1824.909	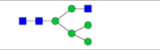
11	1835.216	1835.925	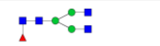
12	1906.198	1906.962	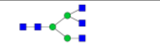
13	1968.199	1968.988	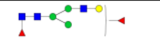
14	1987.171	1987.982	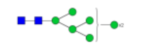
15	2040.180	2040.025	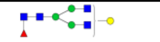
16	2070.134	2070.035	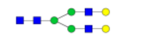
17	2111.098	2111.062	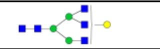
18	2143.112	2143.077	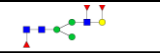
19	2156.094	2156.072	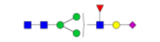
20	2192.835	2192.082	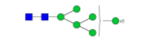
21	2244.094	2244.125	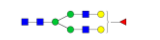
22	2285.971	2285.151	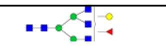
23	2326.249	2326.177	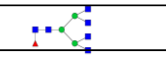
24	2330.036	2330.161	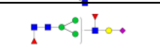
25	2395.988	2396.182	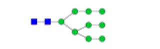
26	2401.015	2401.198	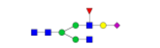
27	2418.229	2418.213	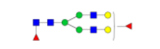
28	2431.970	2431.209	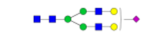
29	2489.912	2489.251	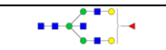
30	2530.337	2530.277	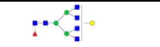
31	2562.967	2562.292	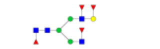
32	2605.951	2605.198	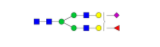
33	2693.430	2693.350	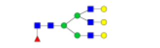
34	2734.467	2734.377	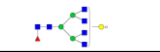
35	2779.723	2779.387	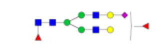
36	2792.865	2792.383	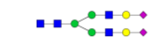

Peak No.	Measured N-glycan Mass [M+Na]$^+$	Theoretical N-glycan Mass [M+Na]$^+$	N-glycan Structure
37	2850.648	2850.424	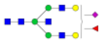
38	2880.636	2880.435	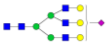
39	2938.584	2938.476	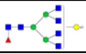
40	2953.589	2953.477	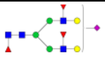
41	2966.593	2966.472	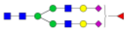
42	3024.570	3024.514	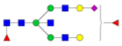
43	3054.565	3054.524	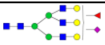
44	3142.495	3142.577	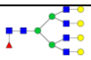
45	3211.478	3211.598	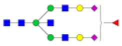
46	3228.435	3228.613	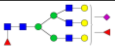
47	3316.398	3316.666	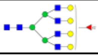
48	3329.360	3329.661	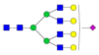
49	3387.316	3387.703	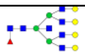
50	3402.338	3402.703	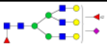
51	3415.282	3415.698	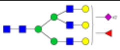
52	3562.432	3561.792	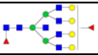
53	3576.243	3576.792	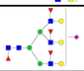
54	3677.112	3677.280	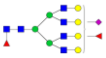
55	3851.036	3851.929	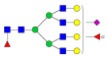
56	4027.004	4026.018	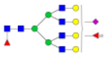
57	4213.011	4213.102	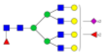
58	4387.109	4387.192	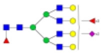
59	4561.164	4561.281	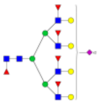
60	4748.251	4748.365	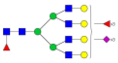
61	4922.237	4922.455	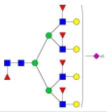
