# Supplementary material for: Glycomic profiling of carcinoembryonic antigen isolated from human tumor tissue
Source: Clin Proteomics. 2015 Jun 27;12(1):17. doi: 10.1186/s12014-015-9088-3 (PMC4495800; doi:10.1186/s12014-015-9088-3)
Supplement: Additional file 2: — Supporting Information (Figures S1-S61). [file 12014_2015_9088_MOESM2_ESM.pdf]

Supporting Information

Glycomic Profiling of Carcinoembryonic Antigen Isolated from Human Tumor Tissue

Chuncui Huang[1] ,(huangchuncui111@163.com)

Tiancheng Zhan[2], (jessecrash@sina.com)

Yaming Liu[1], (lymzzu2009@163.com)

Qianqian Li[1], (liqianqian.2006@163.com)

Hongmei Wu[1],(wuhongmei@moon.ibp.ac.cn)

Dengbo Ji[2], (jidengbo@bjmu.edu.cn)

Yan Li[1],* Correspondence: (yanli@ibp.ac.cn)

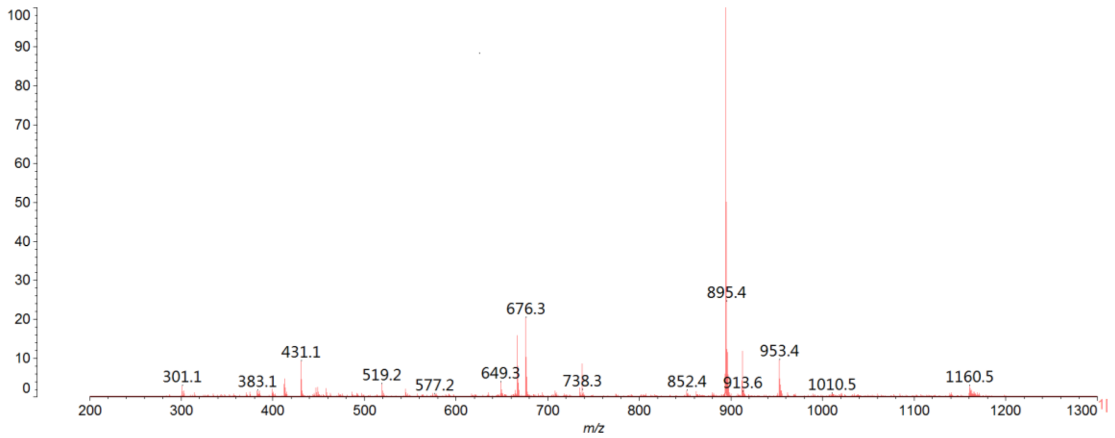

Figure S1, MALDI–TOF/TOF fragment ion spectra of the parent ions at m /z 1171

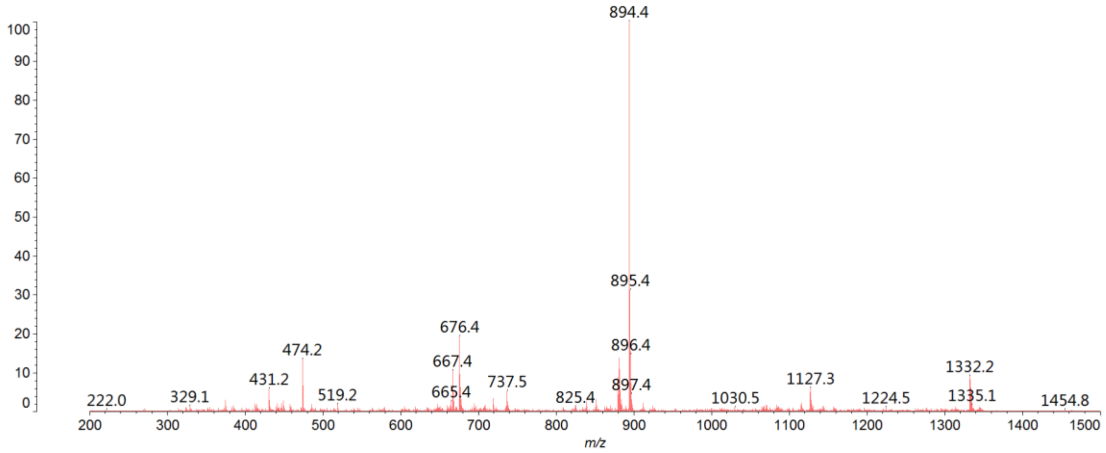

Figure S2, MALDI–TOF/TOF fragment ion spectra of the parent ions at m /z 1345

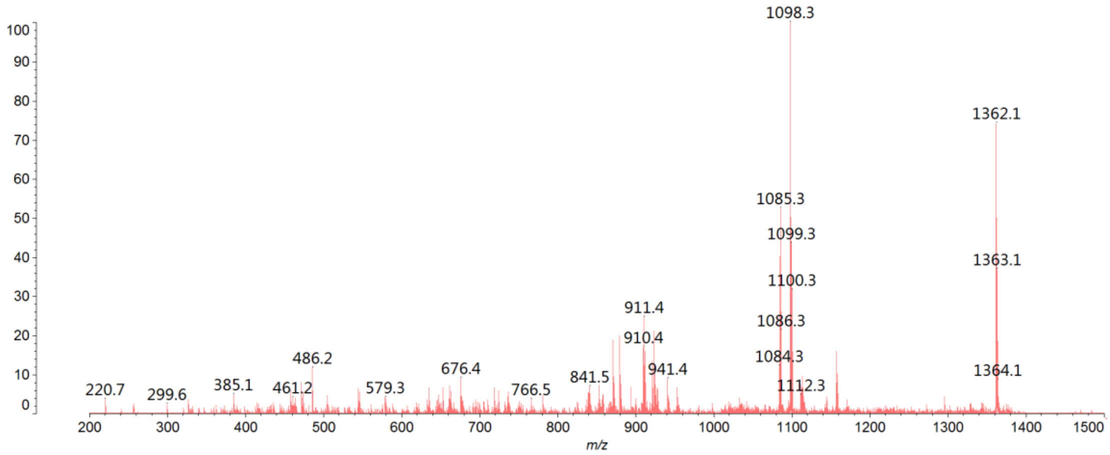

Figure S3, MALDI–TOF/TOF fragment ion spectra of the parent ions at m /z 1375

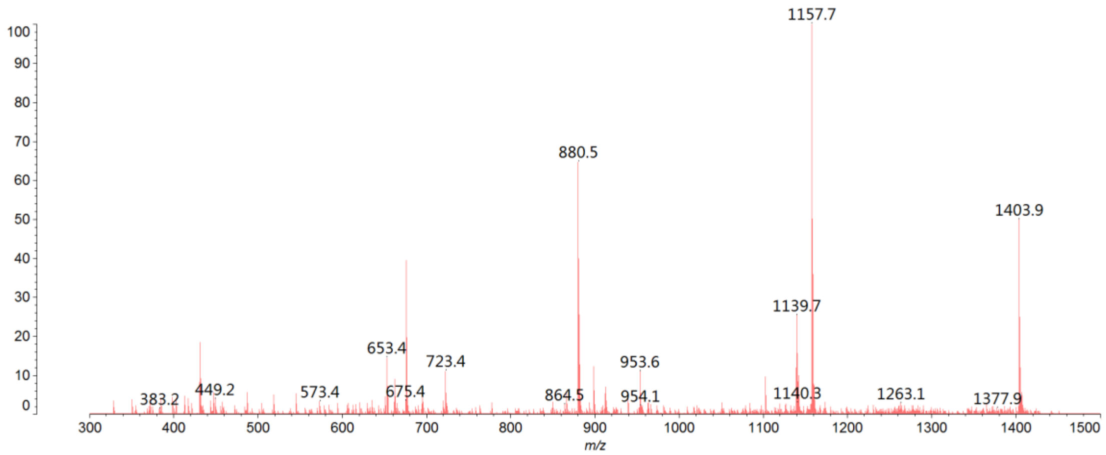

Figure S4, MALDI–TOF/TOF fragment ion spectra of the parent ions at m /z 1416

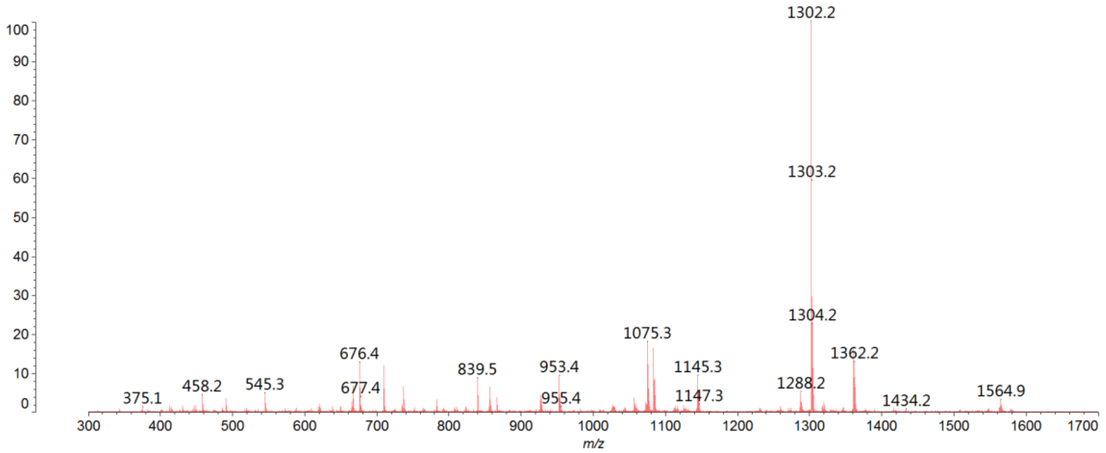

Figure S5, MALDI–TOF/TOF fragment ion spectra of the parent ions at m /z 1579

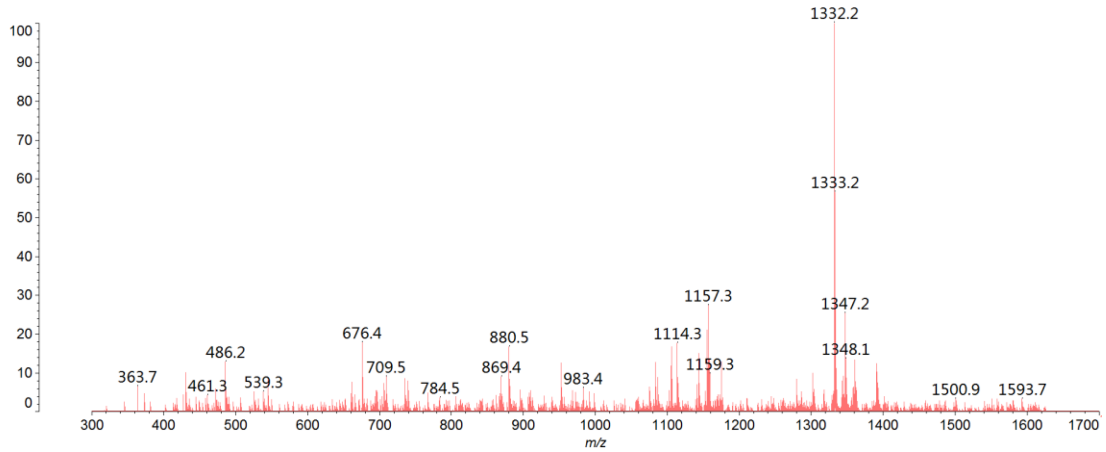

Figure S6, MALDI–TOF/TOF fragment ion spectra of the parent ions at m /z 1620

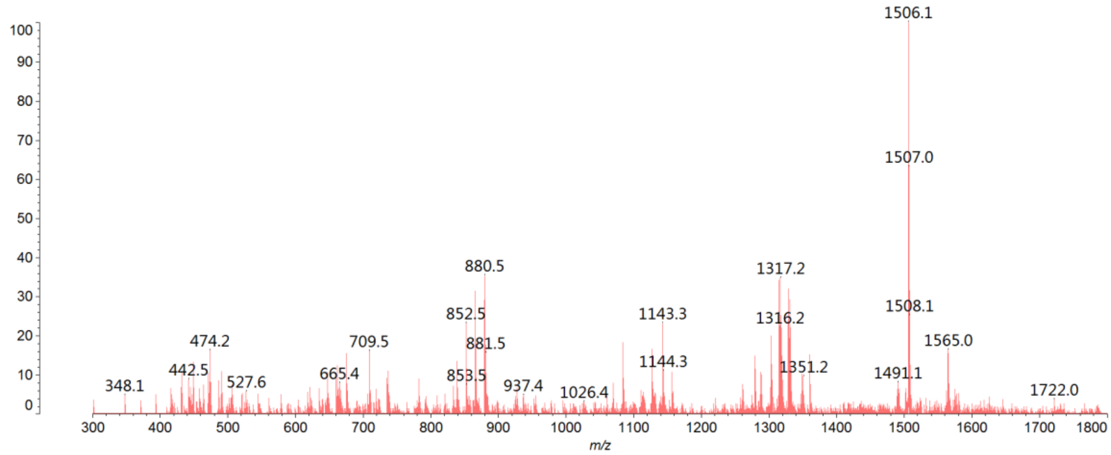

Figure S7, MALDI–TOF/TOF fragment ion spectra of the parent ions at m /z 1783

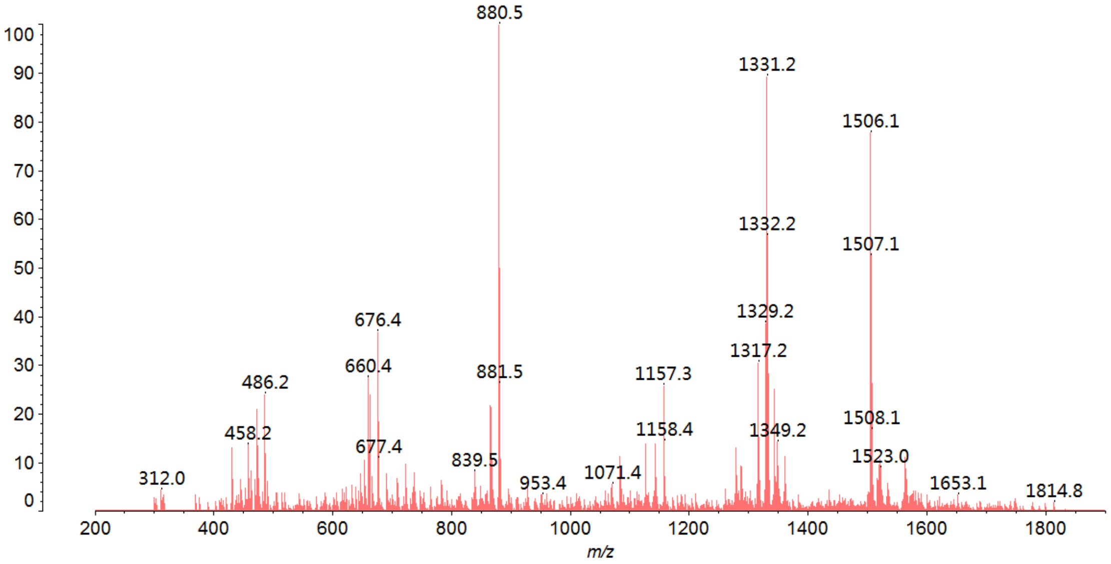

Figure S8, MALDI–TOF/TOF fragment ion spectra of the parent ions at m /z 1794

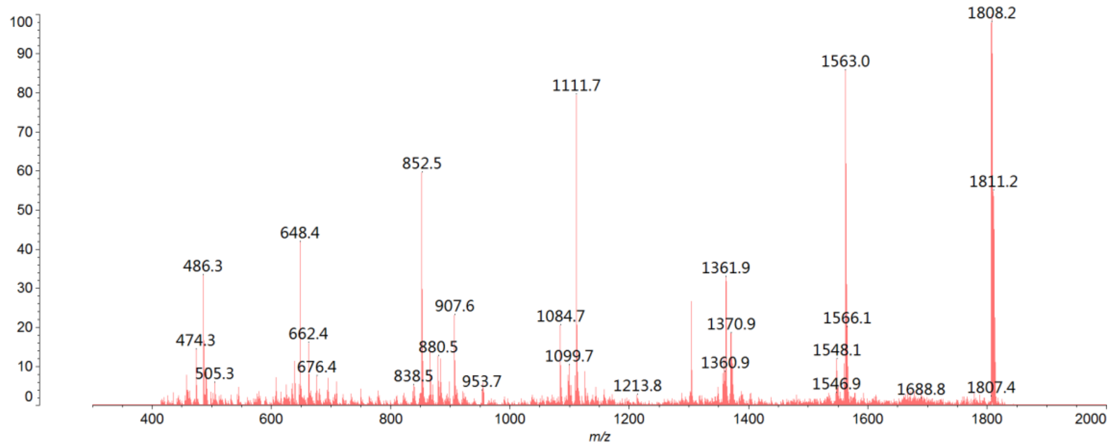

Figure S9, MALDI–TOF/TOF fragment ion spectra of the parent ions at m /z 1824

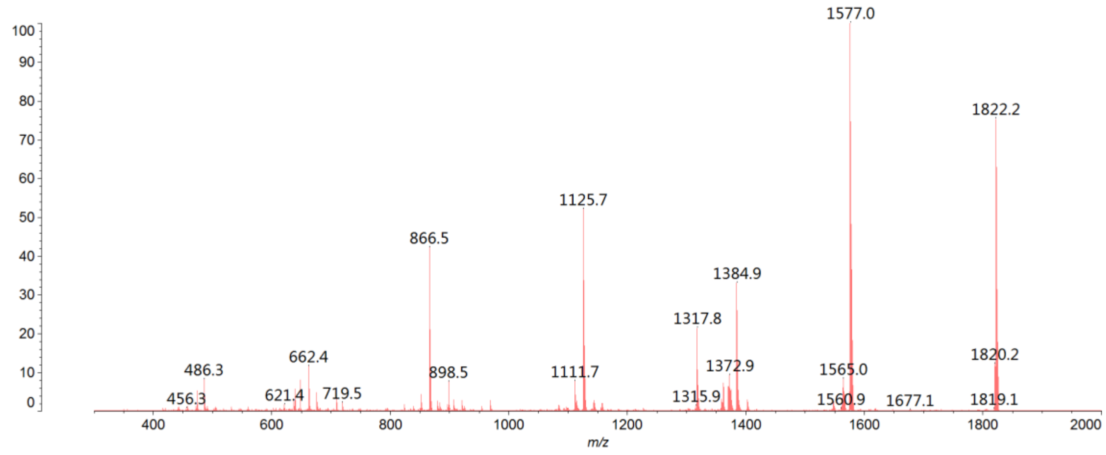

Figure S10, MALDI–TOF/TOF fragment ion spectra of the parent ions at m /z 1835

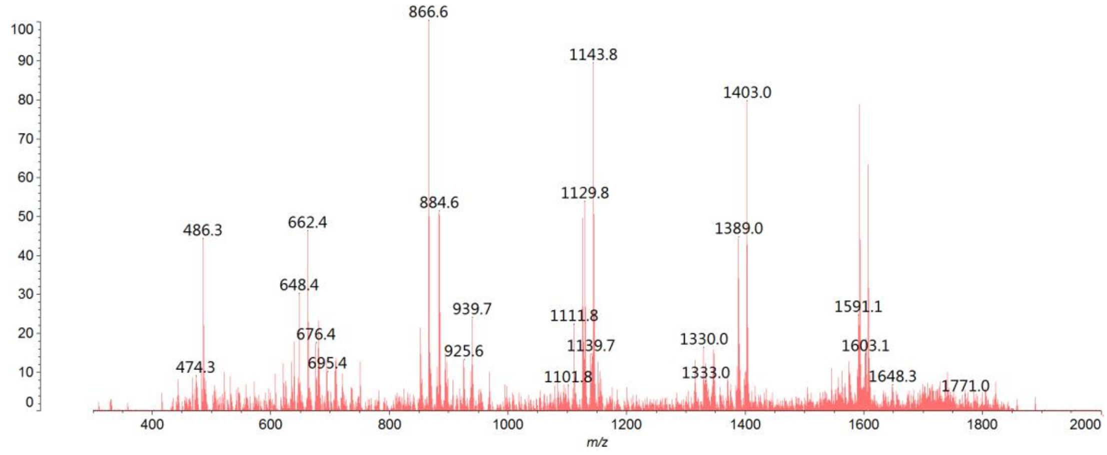

Figure S11, MALDI–TOF/TOF fragment ion spectra of the parent ions at m /z 1866

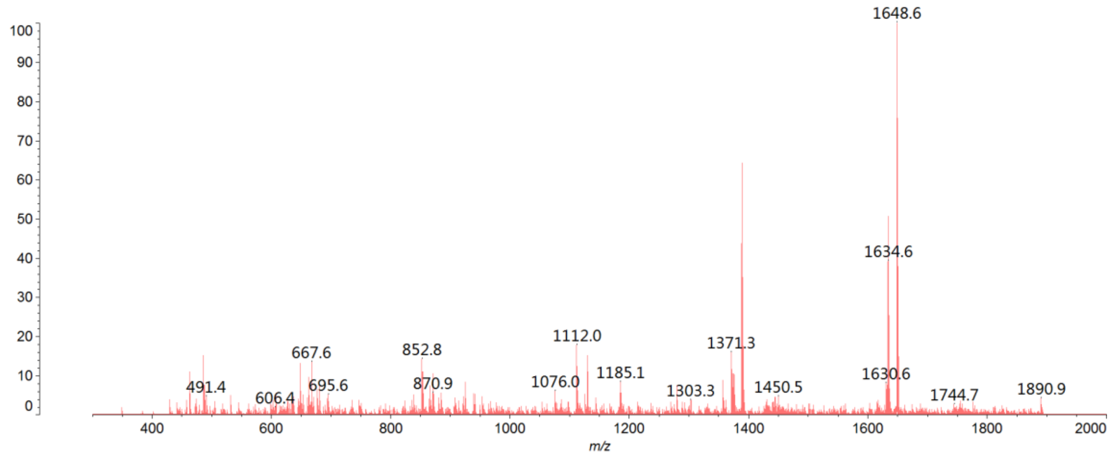

Figure S12, MALDI–TOF/TOF fragment ion spectra of the parent ions at m /z 1906

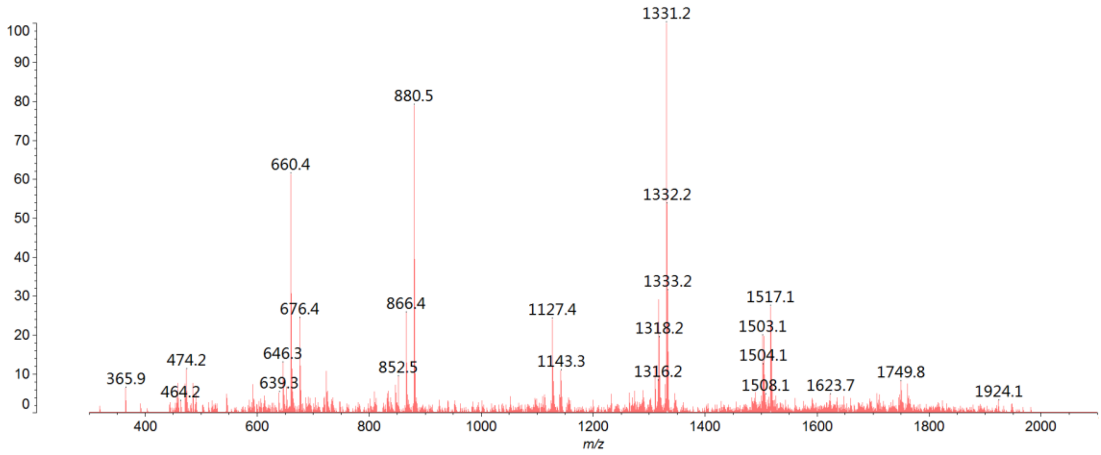

Figure S13, MALDI–TOF/TOF fragment ion spectra of the parent ions at m /z 1968

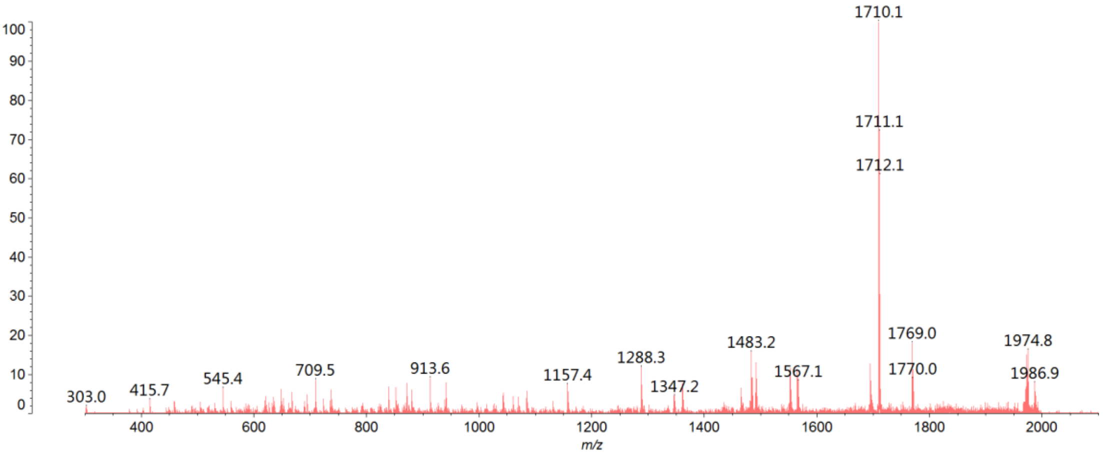

Figure S14, MALDI–TOF/TOF fragment ion spectra of the parent ions at m /z 1987

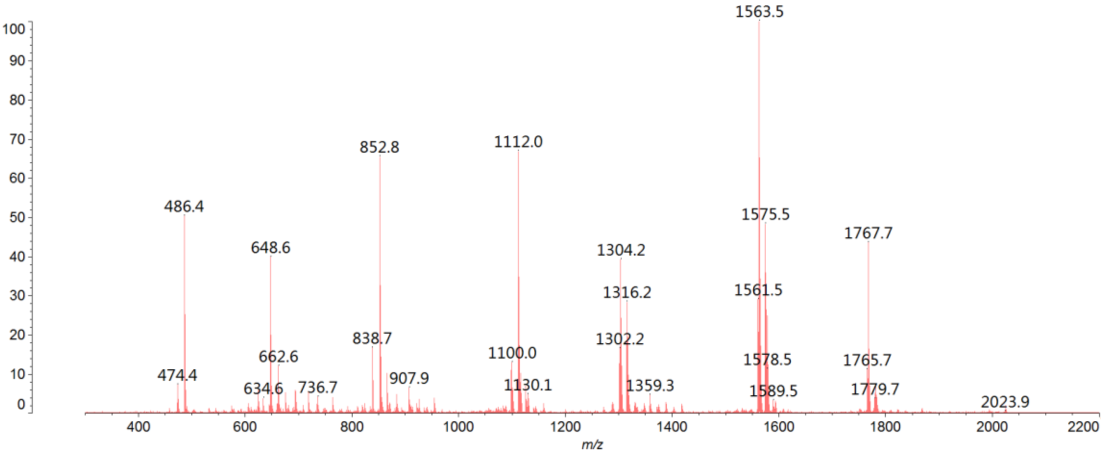

Figure S15, MALDI–TOF/TOF fragment ion spectra of the parent ions at m /z 2040

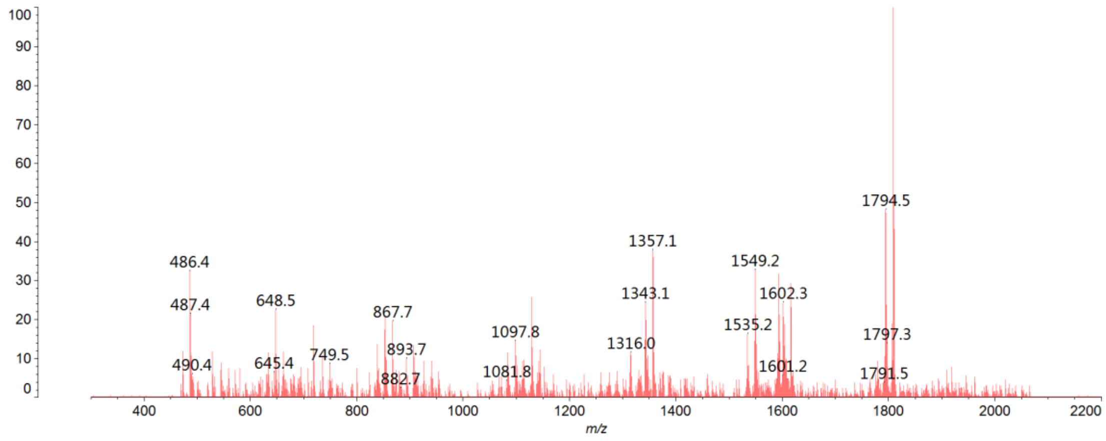

Figure S16, MALDI–TOF/TOF fragment ion spectra of the parent ions at m /z 2070

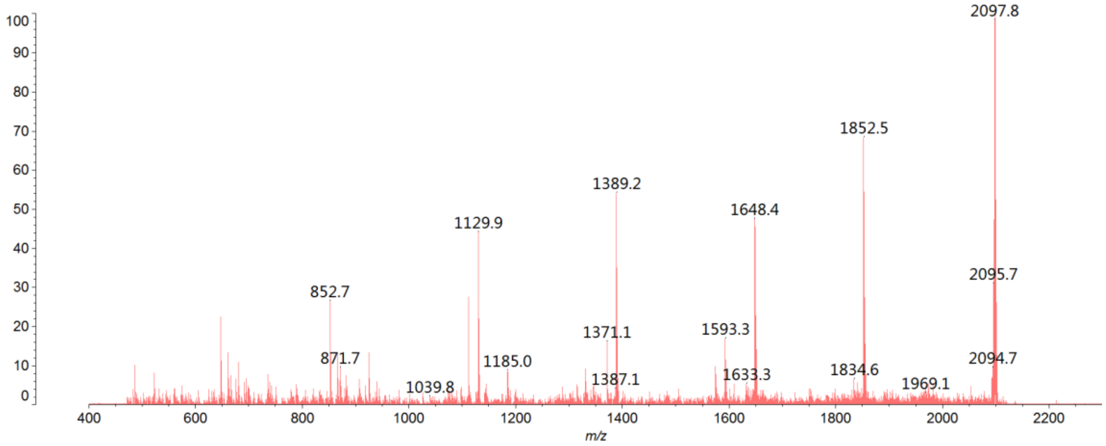

Figure S17, MALDI–TOF/TOF fragment ion spectra of the parent ions at m /z 2111

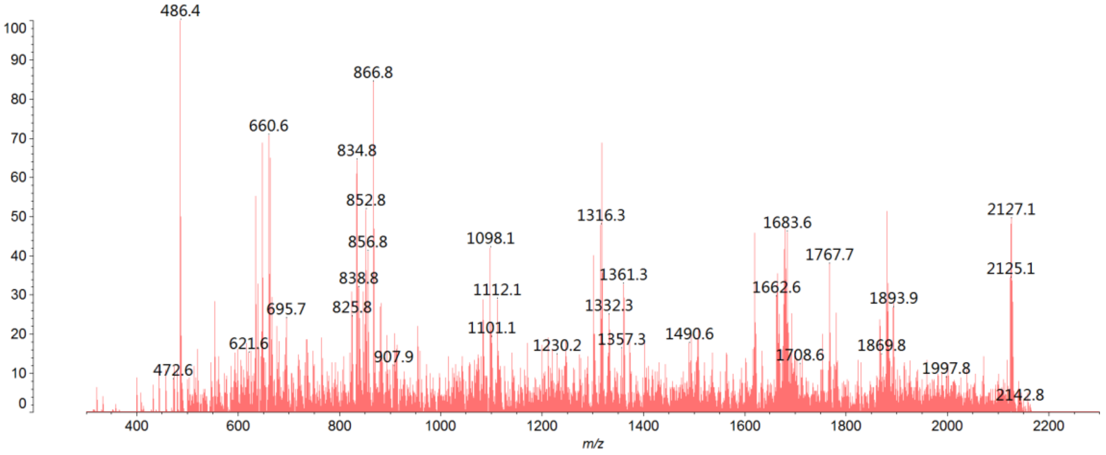

Figure S18, MALDI–TOF/TOF fragment ion spectra of the parent ions at m /z 2143

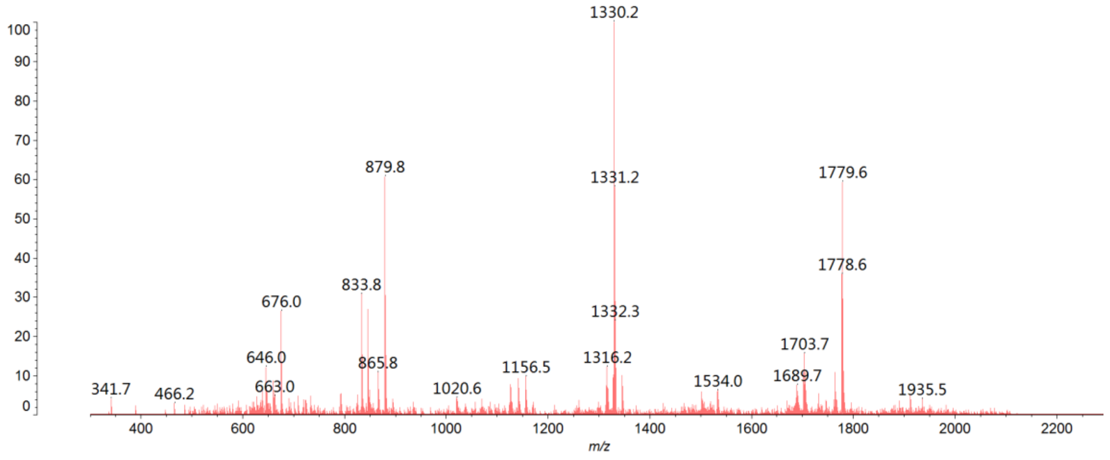

Figure S19, MALDI–TOF/TOF fragment ion spectra of the parent ions at m /z 2156

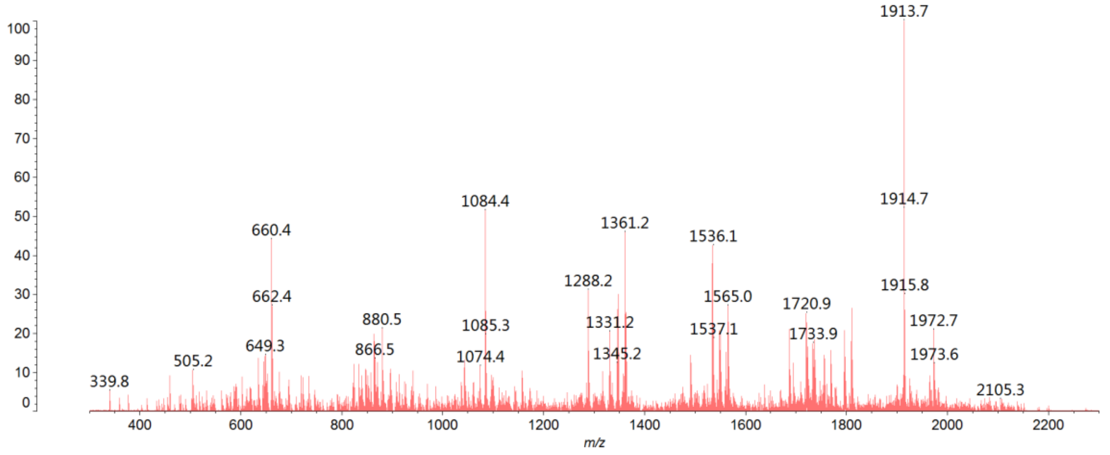

Figure S20, MALDI–TOF/TOF fragment ion spectra of the parent ions at m /z 2192

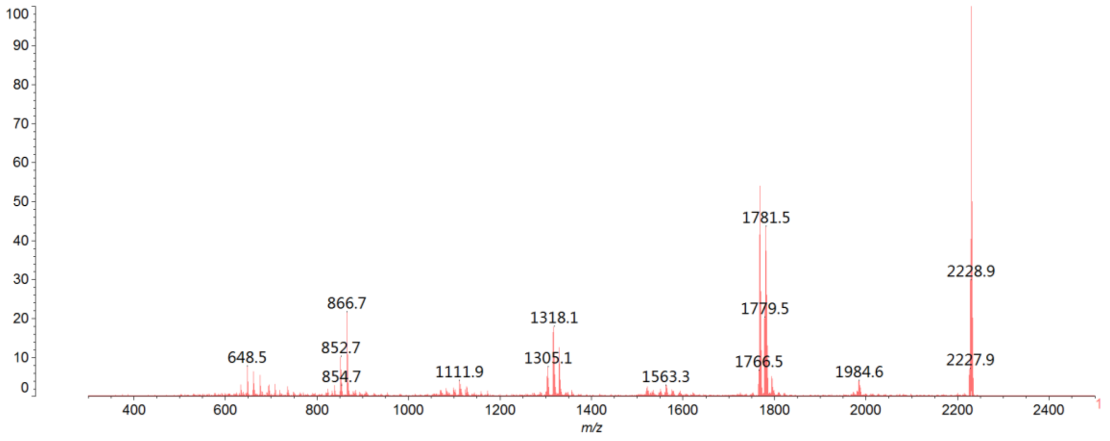

Figure S21, MALDI–TOF/TOF fragment ion spectra of the parent ions at m /z 2244

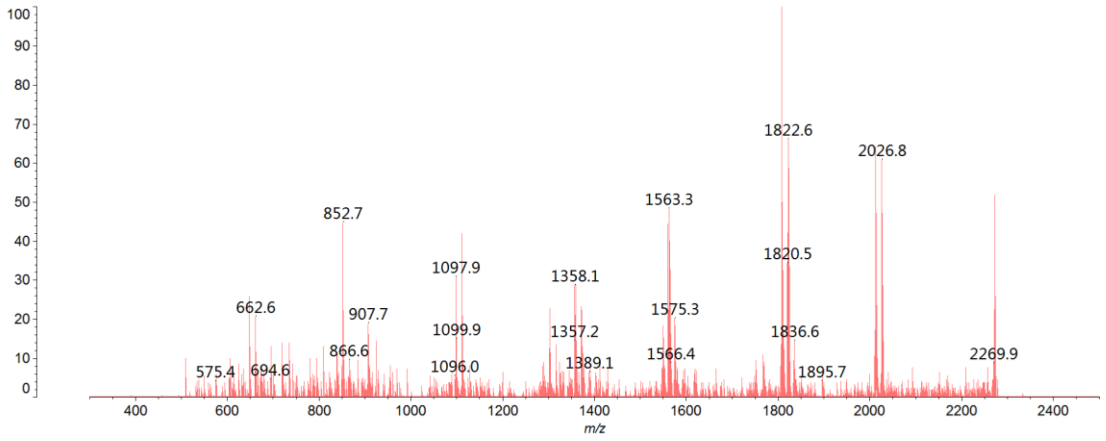

Figure S22, MALDI–TOF/TOF fragment ion spectra of the parent ions at m /z 2285

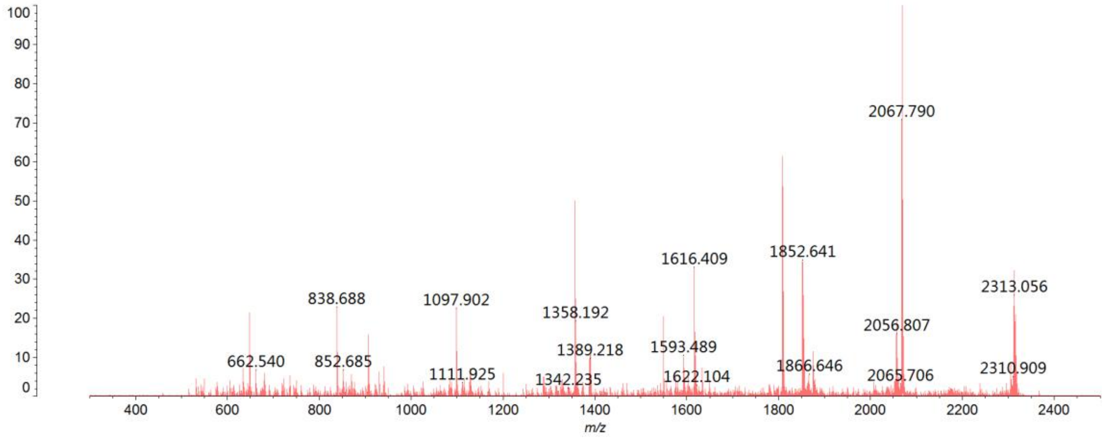

Figure S23, MALDI–TOF/TOF fragment ion spectra of the parent ions at m /z 2326

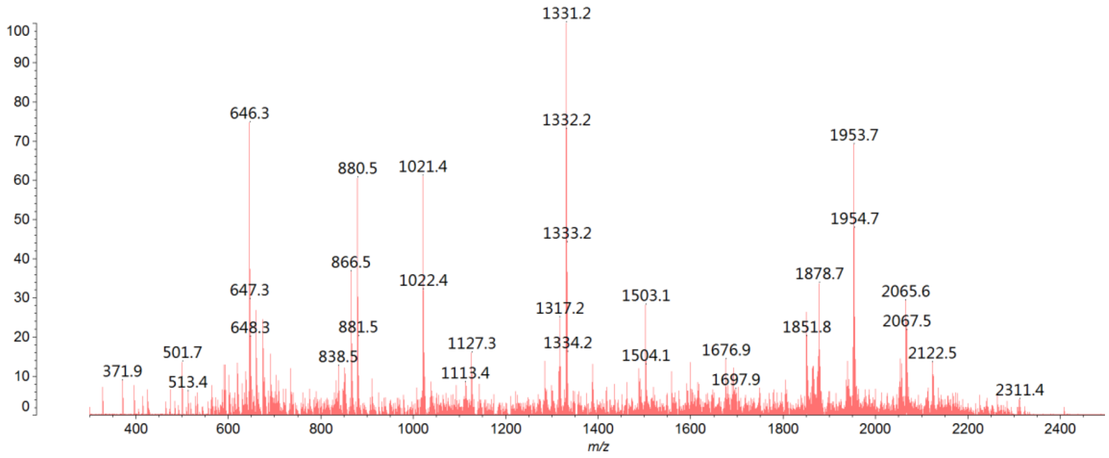

Figure S24, MALDI–TOF/TOF fragment ion spectra of the parent ions at m /z 2330

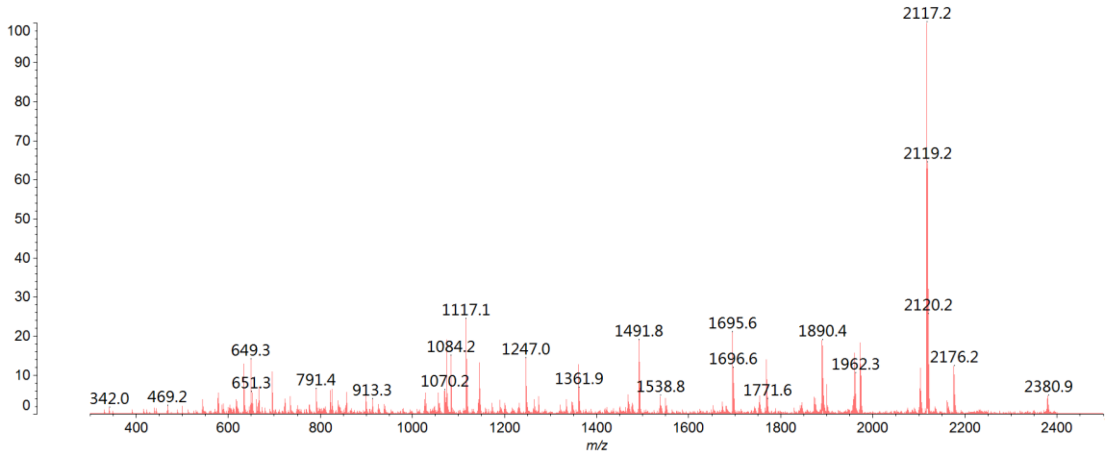

Figure S25, MALDI–TOF/TOF fragment ion spectra of the parent ions at m /z 2395

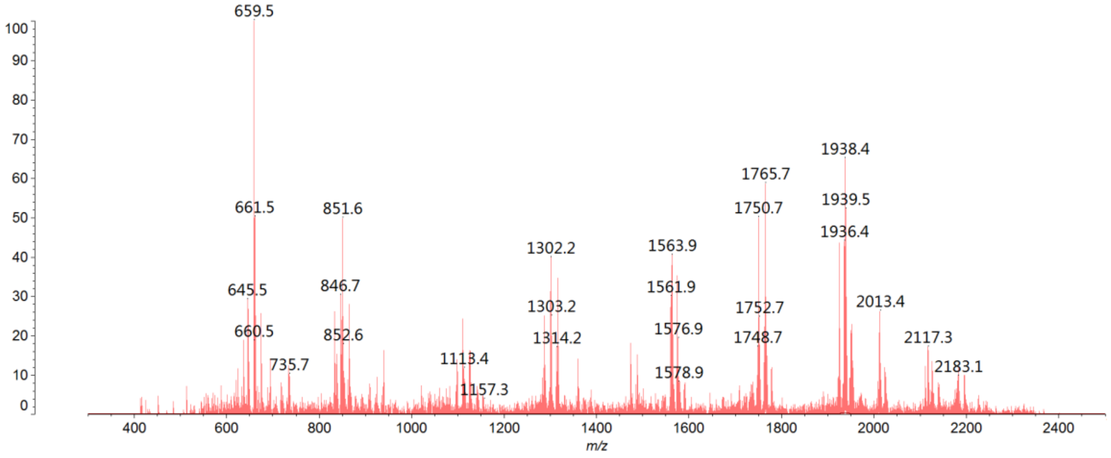

Figure S26, MALDI–TOF/TOF fragment ion spectra of the parent ions at m /z 2401

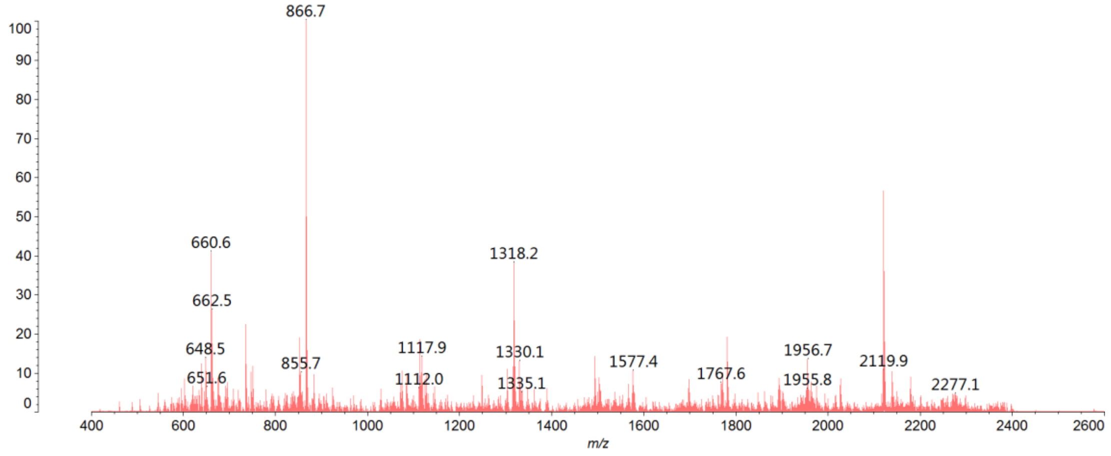

Figure S27, MALDI–TOF/TOF fragment ion spectra of the parent ions at m /z 2418

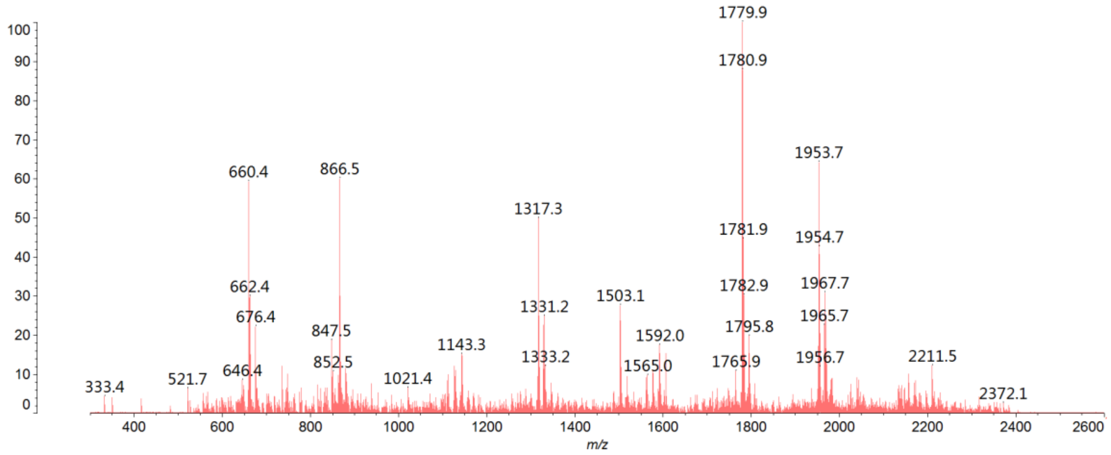

Figure S28, MALDI–TOF/TOF fragment ion spectra of the parent ions at m /z 2431

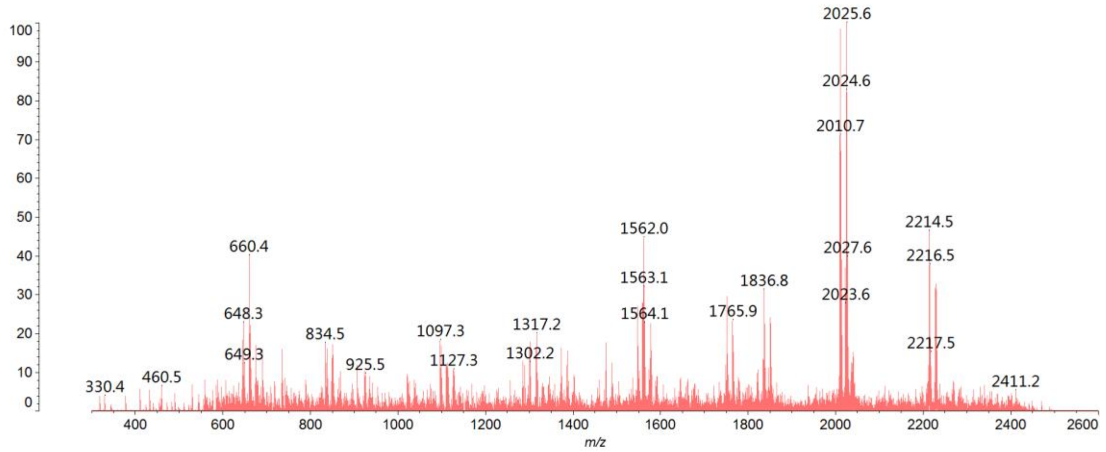

Figure S29, MALDI–TOF/TOF fragment ion spectra of the parent ions at m /z 2489

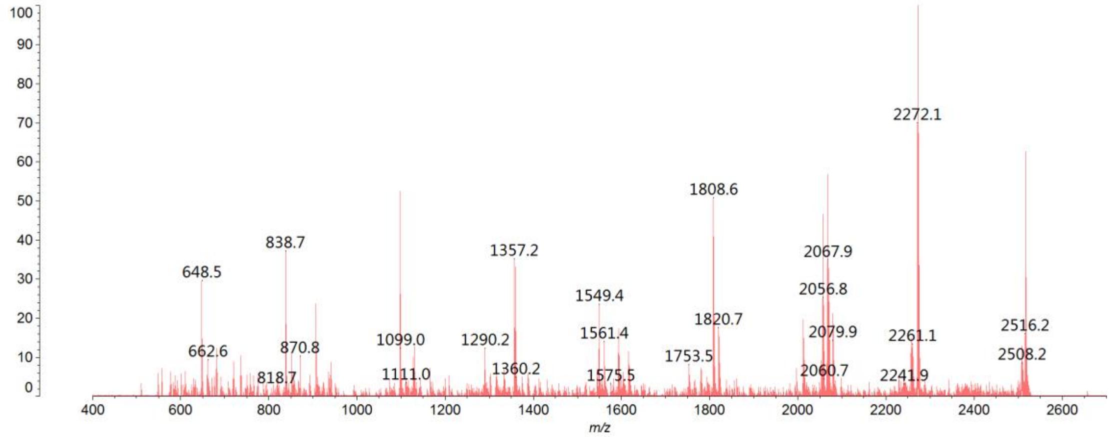

Figure S30, MALDI–TOF/TOF fragment ion spectra of the parent ions at m /z 2530

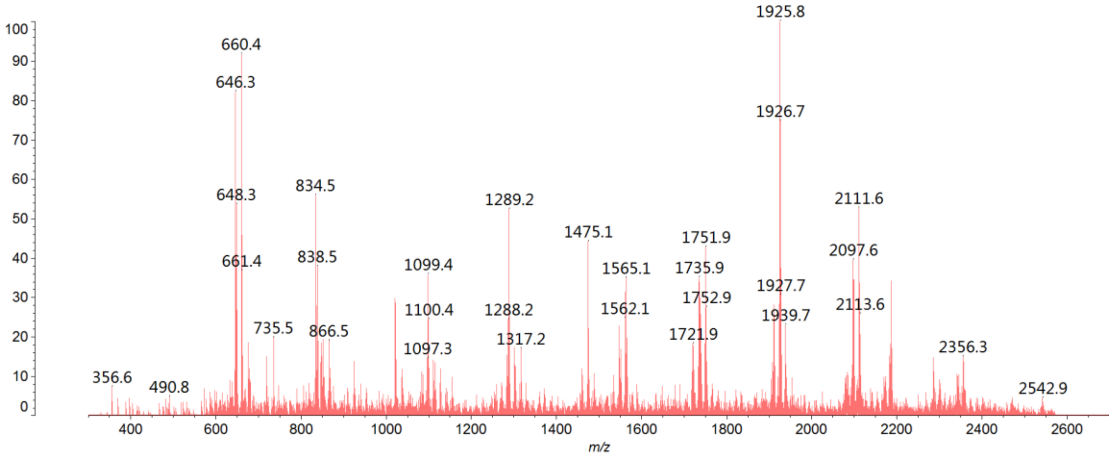

Figure S31, MALDI–TOF/TOF fragment ion spectra of the parent ions at m /z 2562

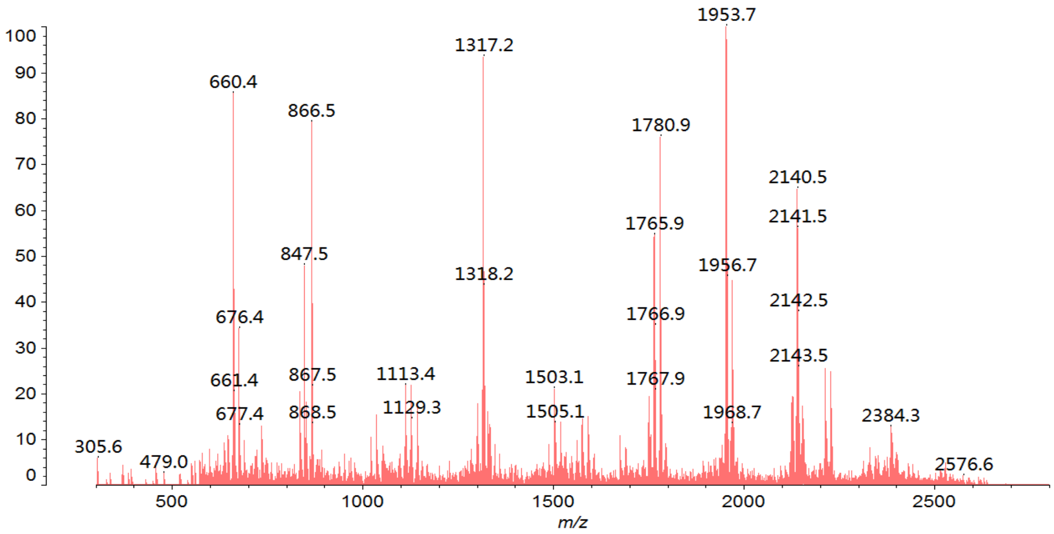

Figure S32, MALDI–TOF/TOF fragment ion spectra of the parent ions at m /z 2605

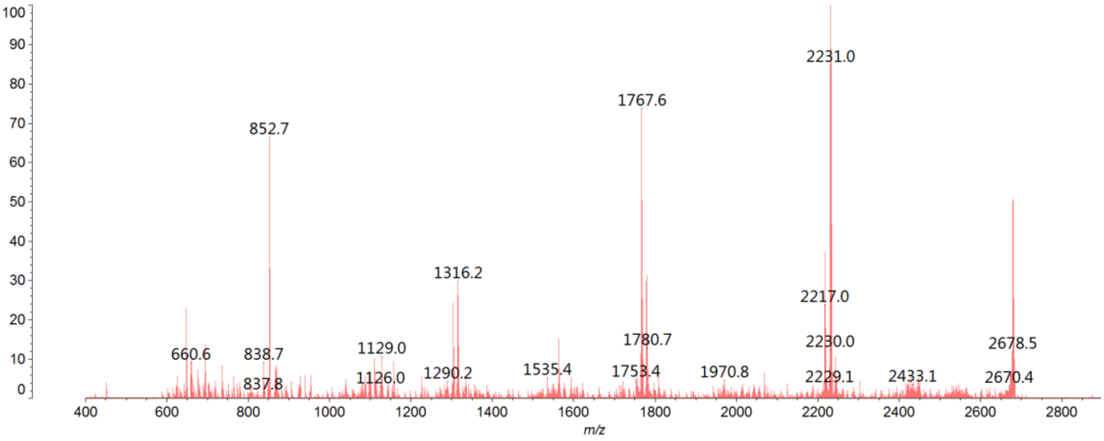

Figure S33, MALDI–TOF/TOF fragment ion spectra of the parent ions at m /z 2693

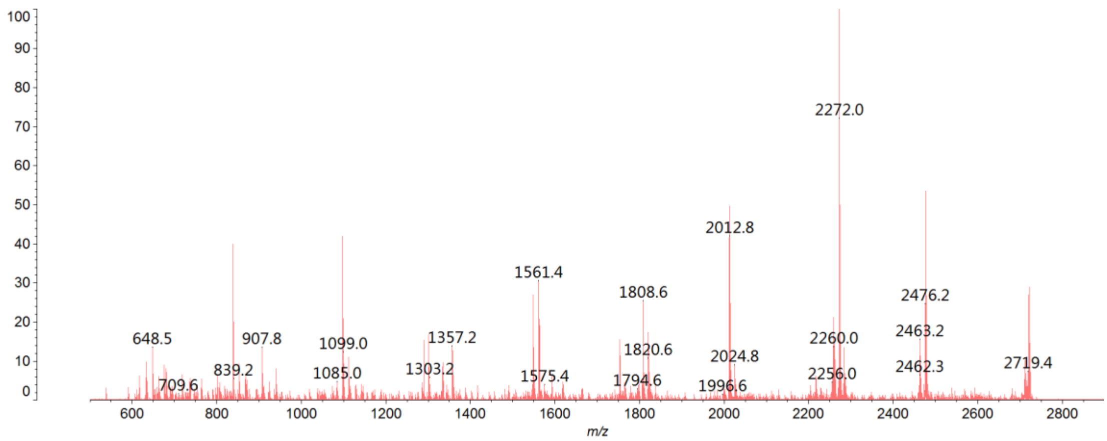

Figure S34, MALDI–TOF/TOF fragment ion spectra of the parent ions at m /z 2734

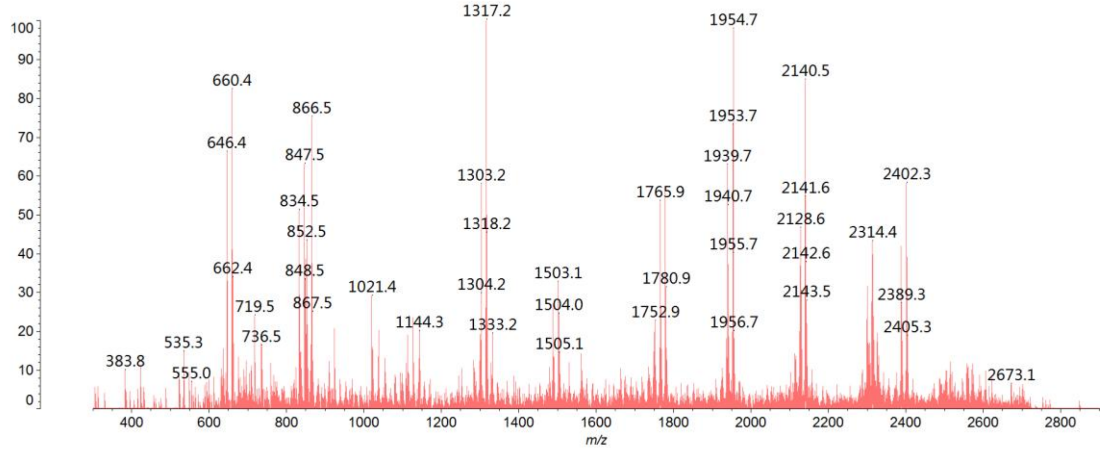

Figure S35, MALDI–TOF/TOF fragment ion spectra of the parent ions at m /z 2779

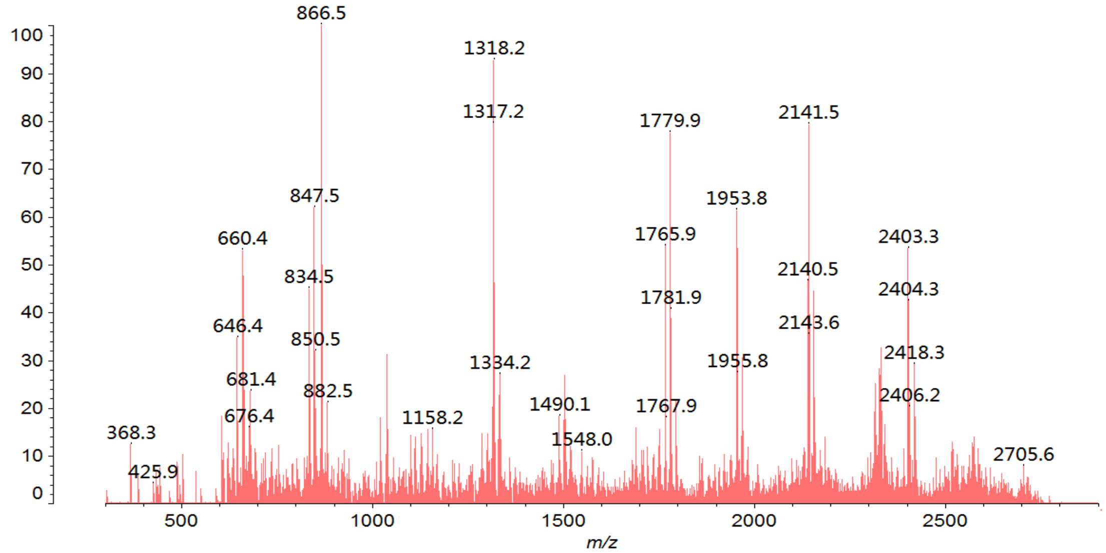

Figure S36, MALDI–TOF/TOF fragment ion spectra of the parent ions at m /z 2792

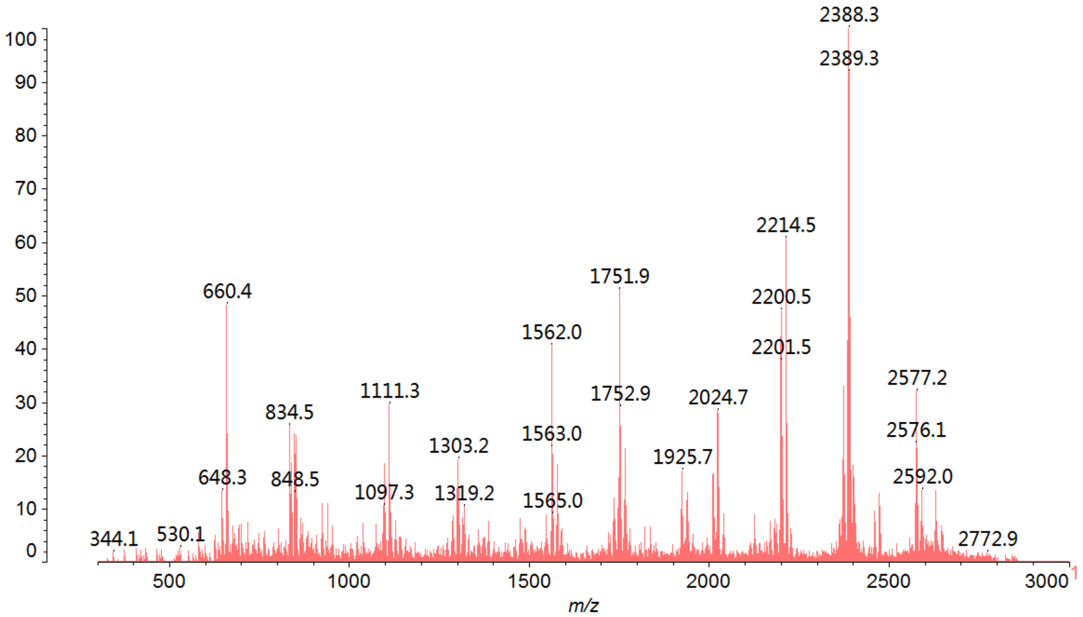

Figure S37, MALDI–TOF/TOF fragment ion spectra of the parent ions at m /z 2850

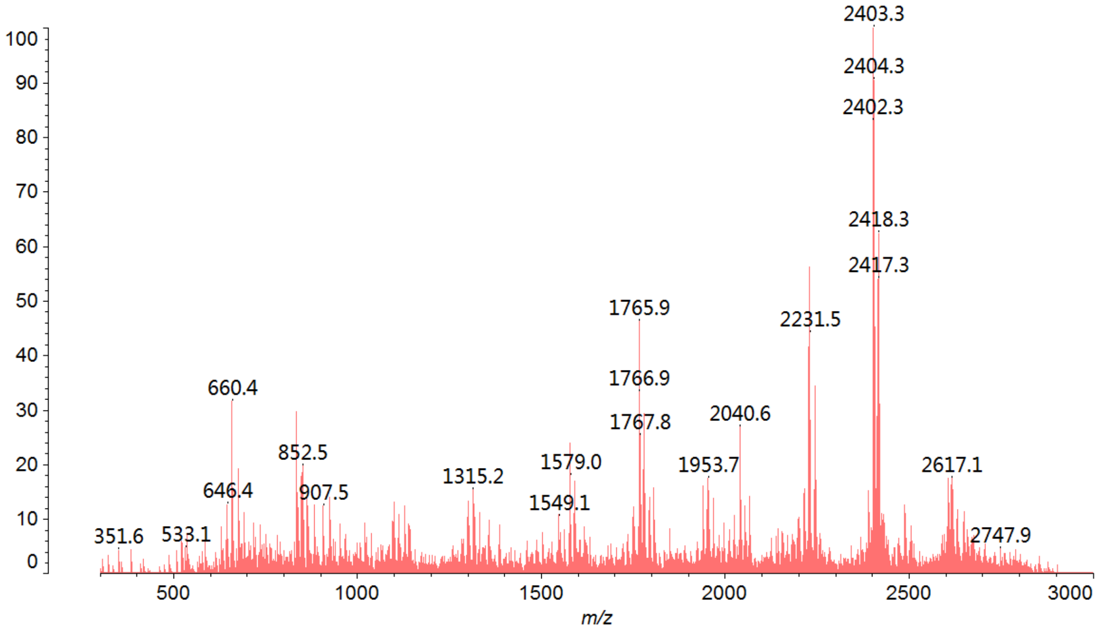

Figure S38, MALDI–TOF/TOF fragment ion spectra of the parent ions at m /z 2880

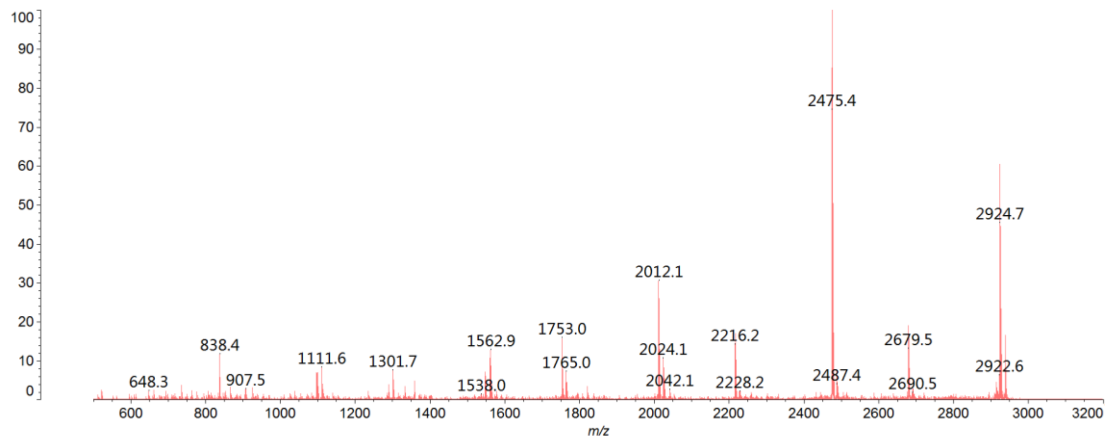

Figure S39, MALDI–TOF/TOF fragment ion spectra of the parent ions at m /z 2938

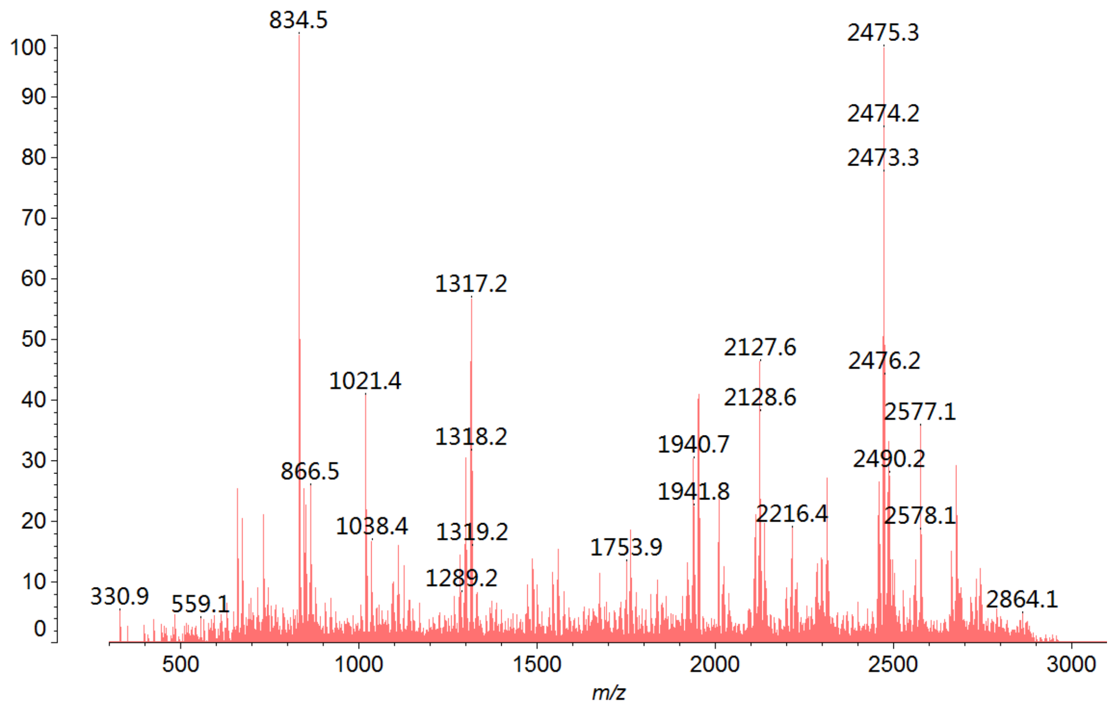

Figure S40, MALDI–TOF/TOF fragment ion spectra of the parent ions at m /z 2953

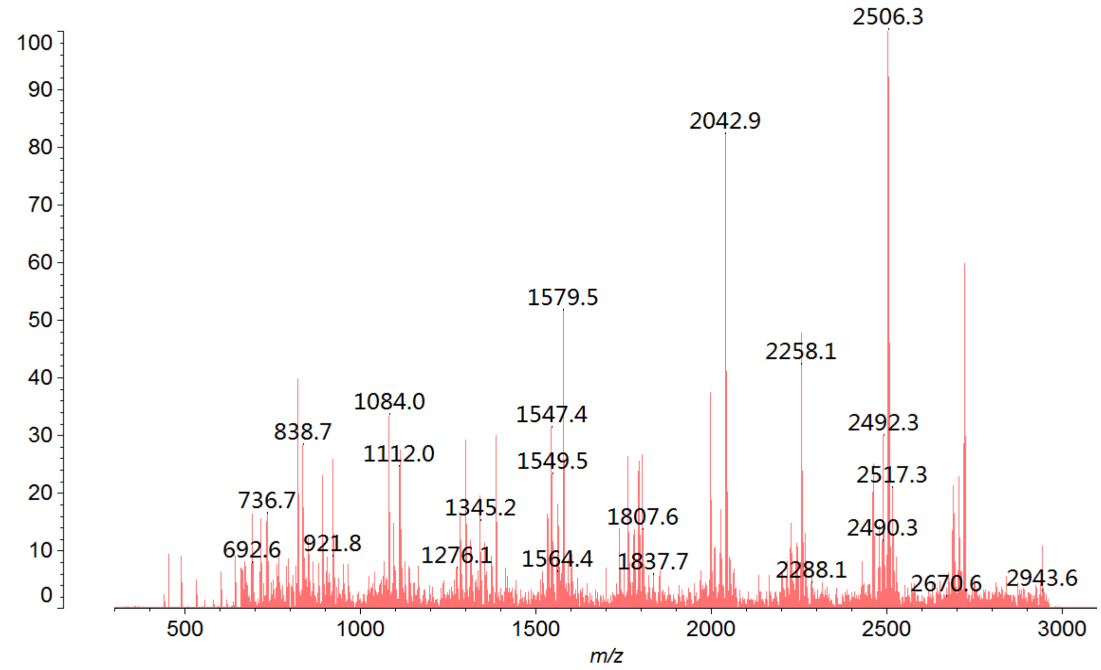

Figure S41, MALDI–TOF/TOF fragment ion spectra of the parent ions at m /z 2966

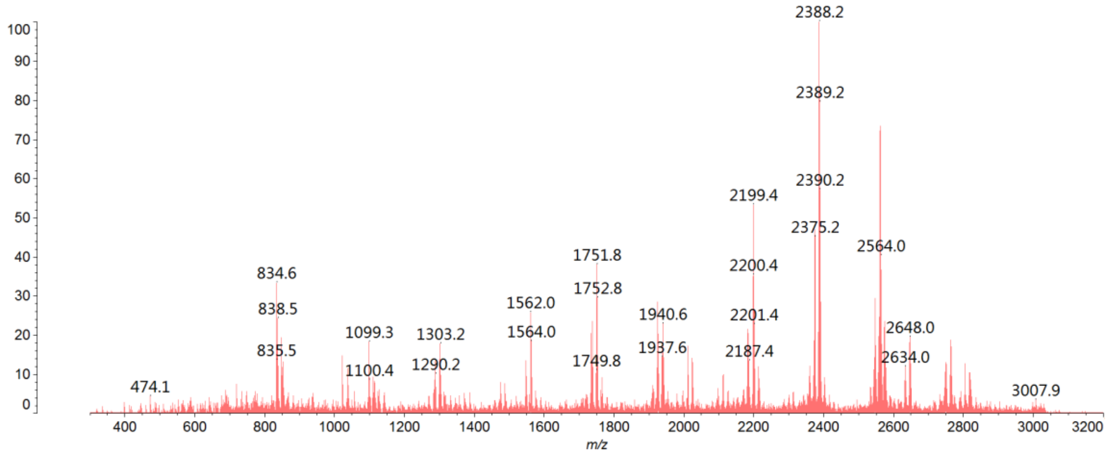

Figure S42, MALDI–TOF/TOF fragment ion spectra of the parent ions at m /z 3024

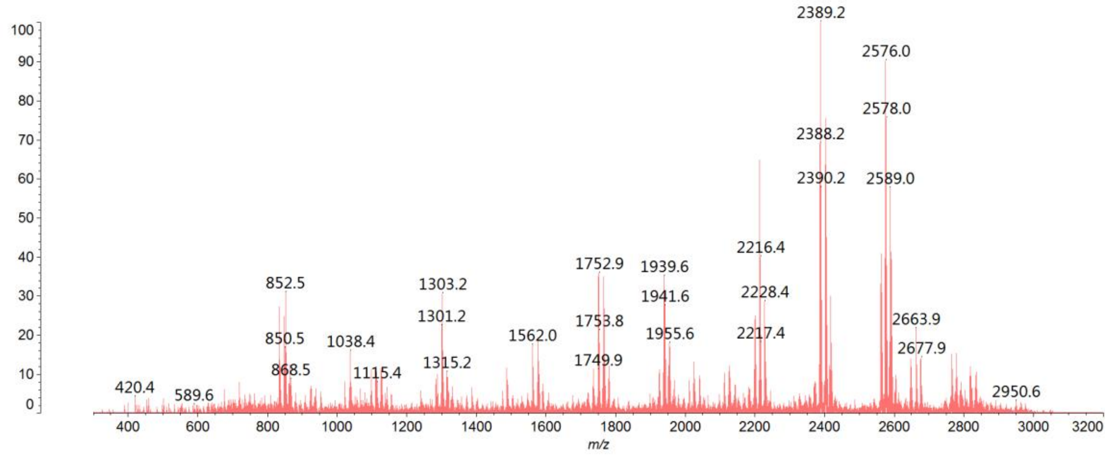

Figure S43, MALDI–TOF/TOF fragment ion spectra of the parent ions at m /z 3054

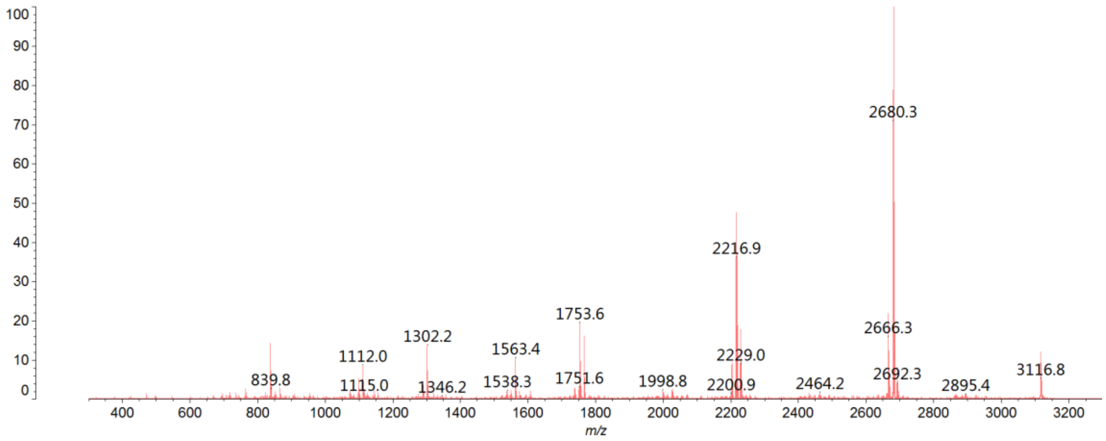

Figure S44, MALDI–TOF/TOF fragment ion spectra of the parent ions at m /z 3142

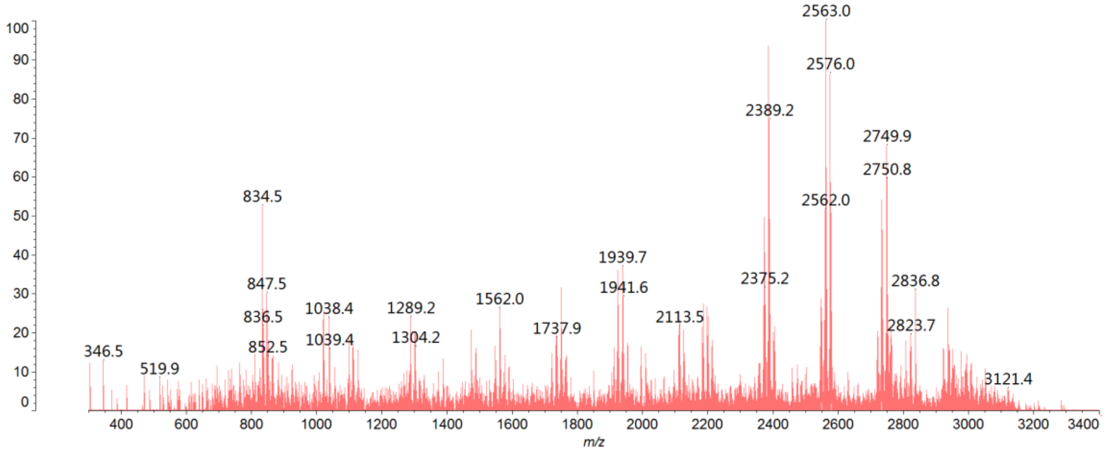

Figure S45, MALDI–TOF/TOF fragment ion spectra of the parent ions at m /z 3211

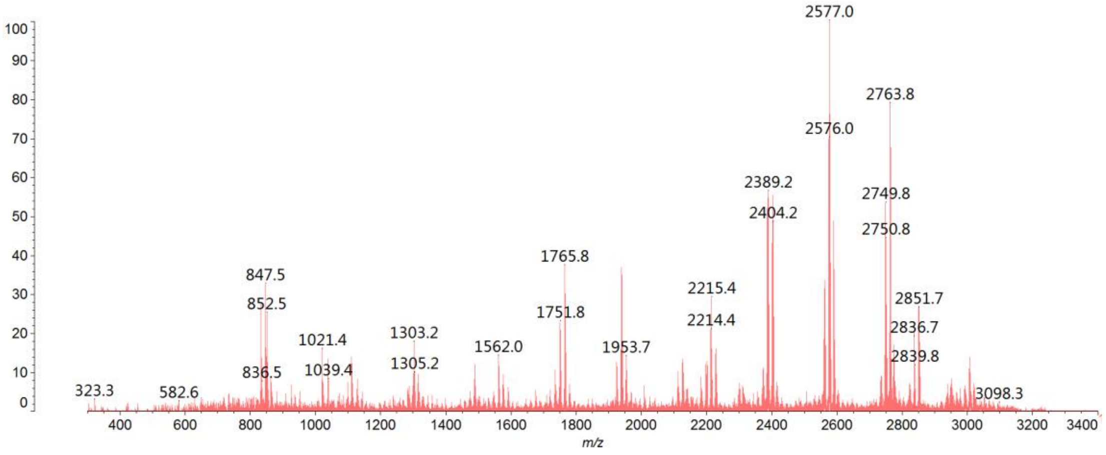

Figure S46, MALDI–TOF/TOF fragment ion spectra of the parent ions at m /z 3228

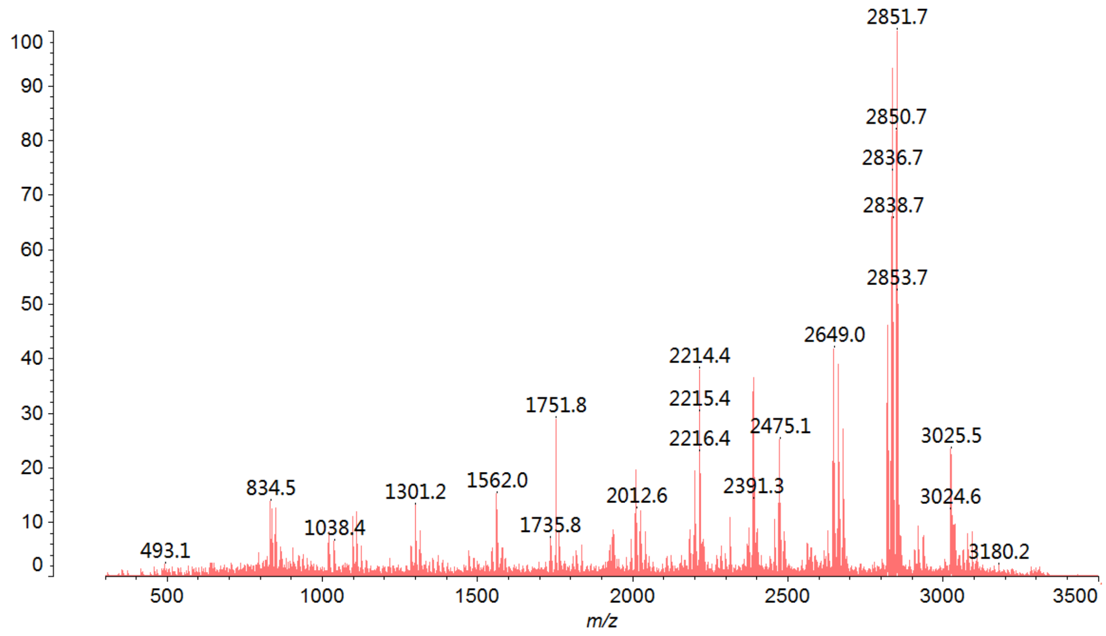

Figure S47, MALDI–TOF/TOF fragment ion spectra of the parent ions at m /z 3316

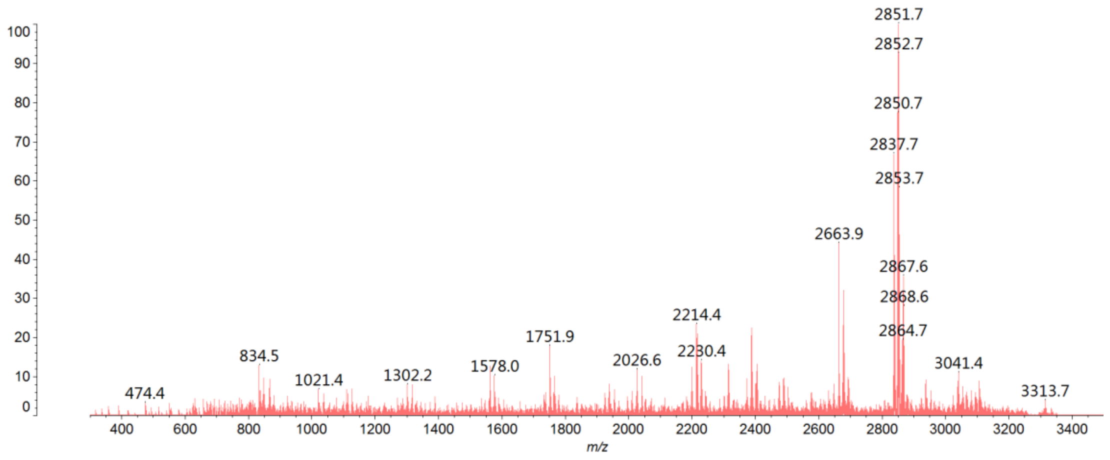

Figure S48, MALDI–TOF/TOF fragment ion spectra of the parent ions at m /z 3329

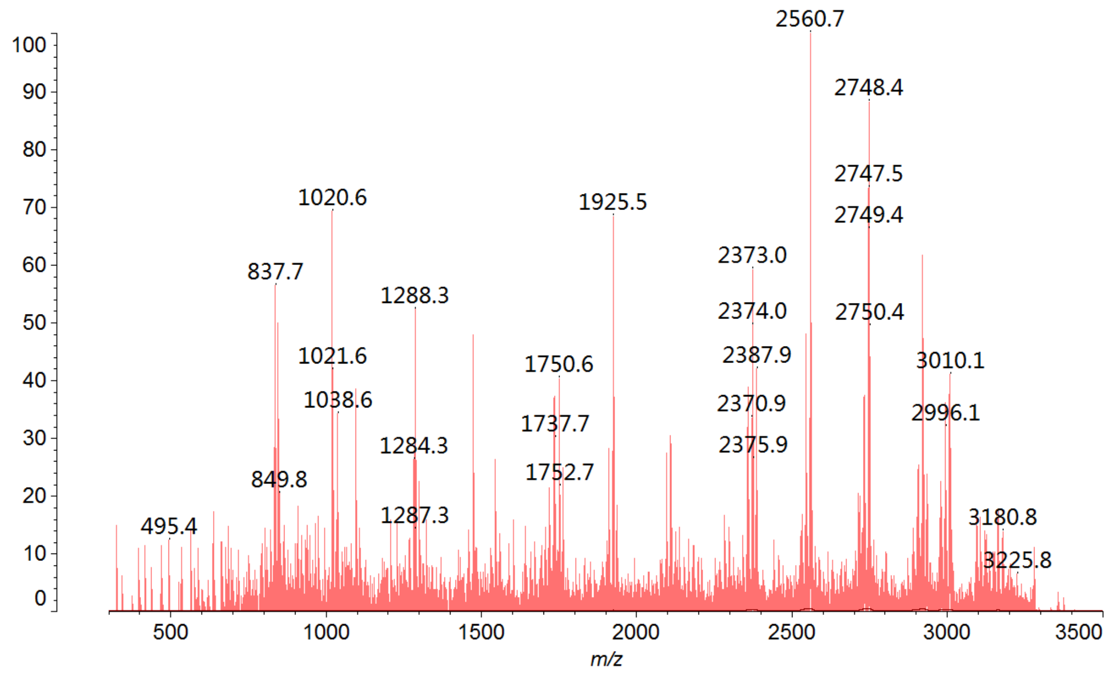

Figure S49, MALDI–TOF/TOF fragment ion spectra of the parent ions at m /z 3387

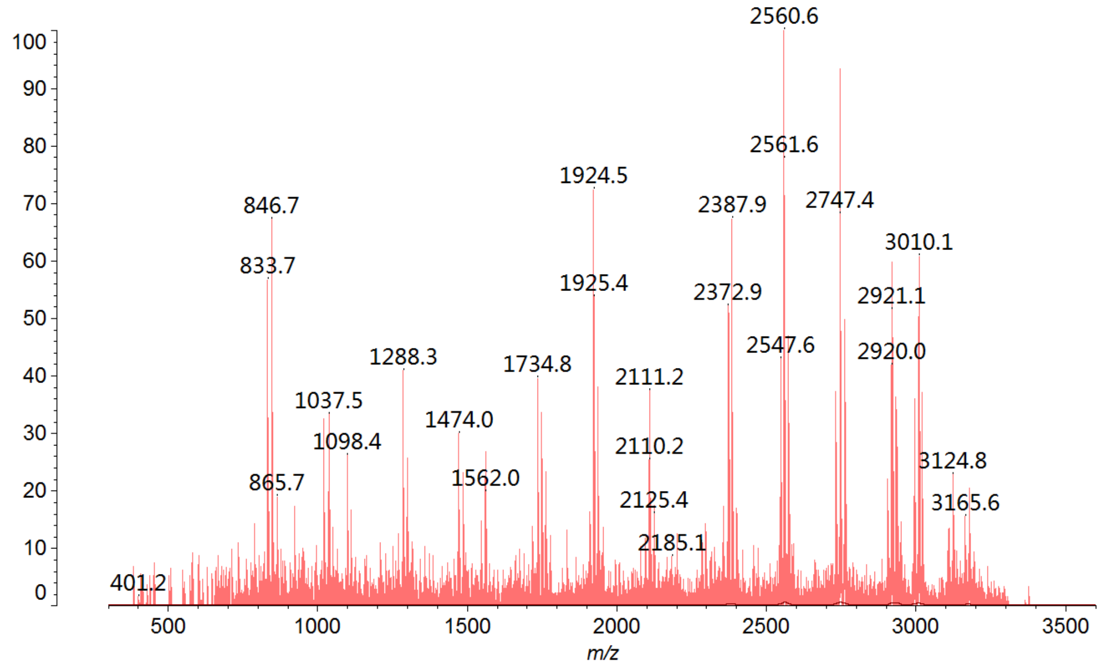

Figure S50, MALDI–TOF/TOF fragment ion spectra of the parent ions at m /z 3402

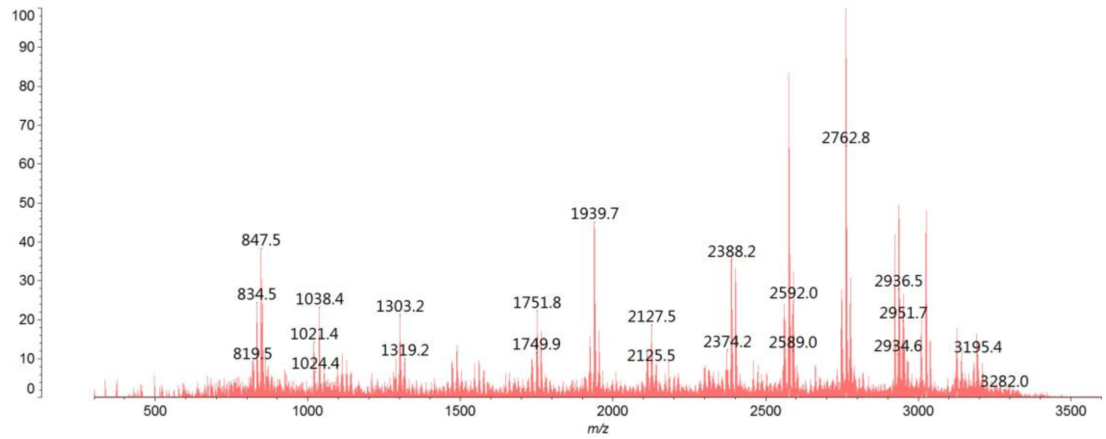

Figure S51, MALDI–TOF/TOF fragment ion spectra of the parent ions at m /z 3415

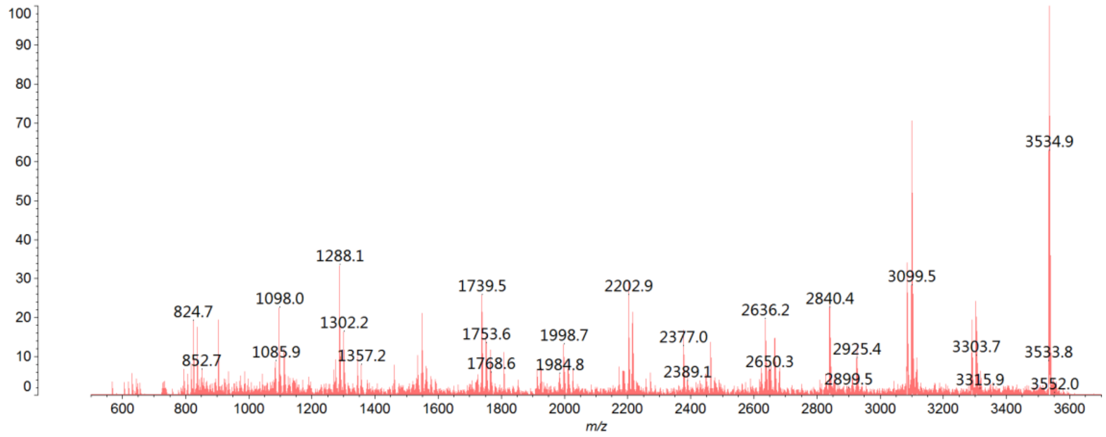

Figure S52, MALDI–TOF/TOF fragment ion spectra of the parent ions at m /z 3562

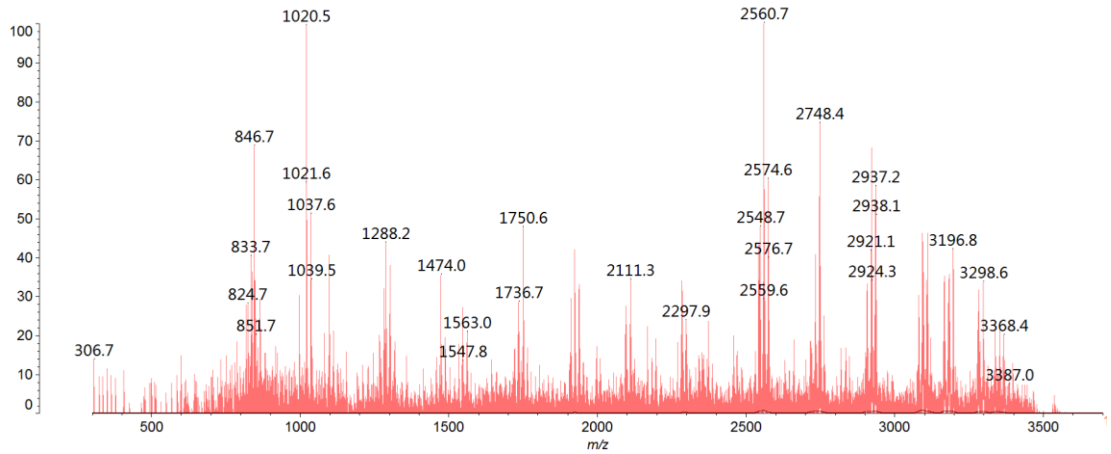

Figure S53, MALDI–TOF/TOF fragment ion spectra of the parent ions at m /z 3576

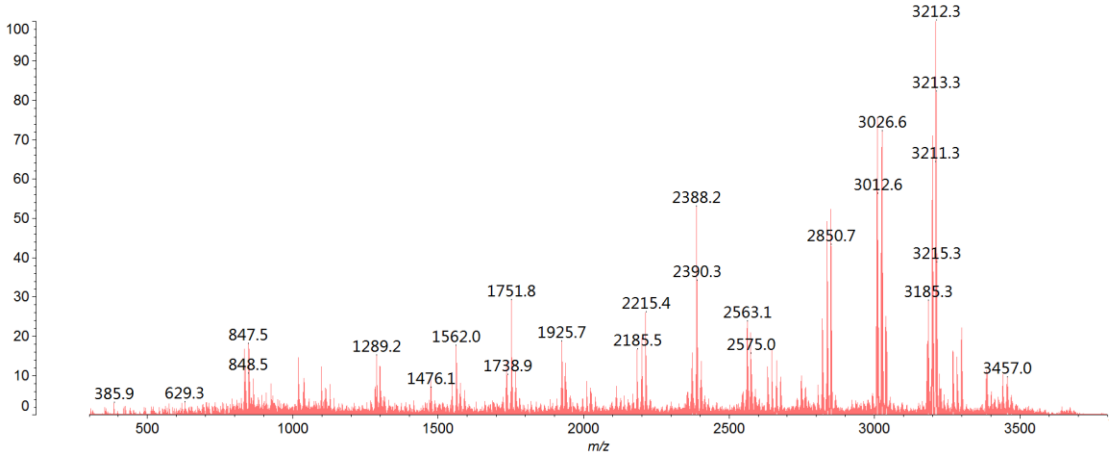

Figure S54, MALDI–TOF/TOF fragment ion spectra of the parent ions at m /z 3677

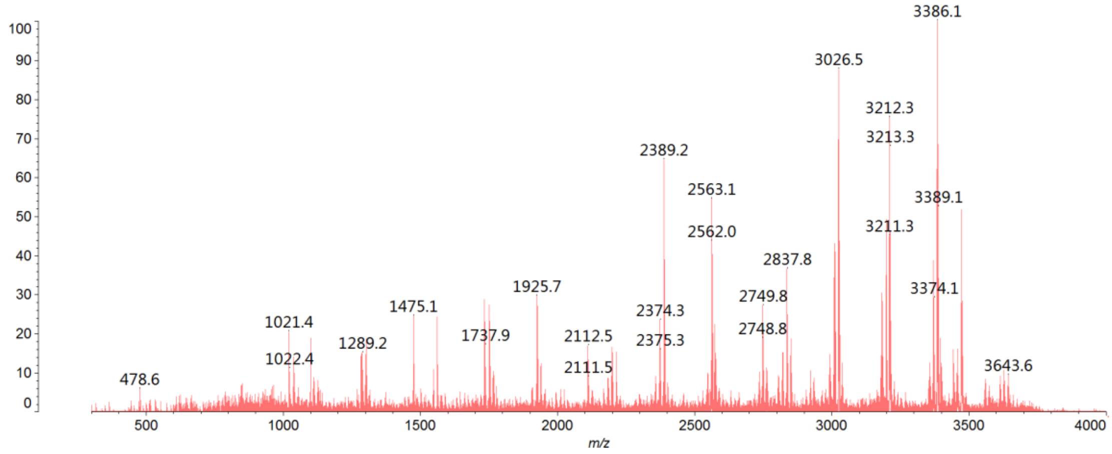

Figure S55, MALDI–TOF/TOF fragment ion spectra of the parent ions at m /z 3851

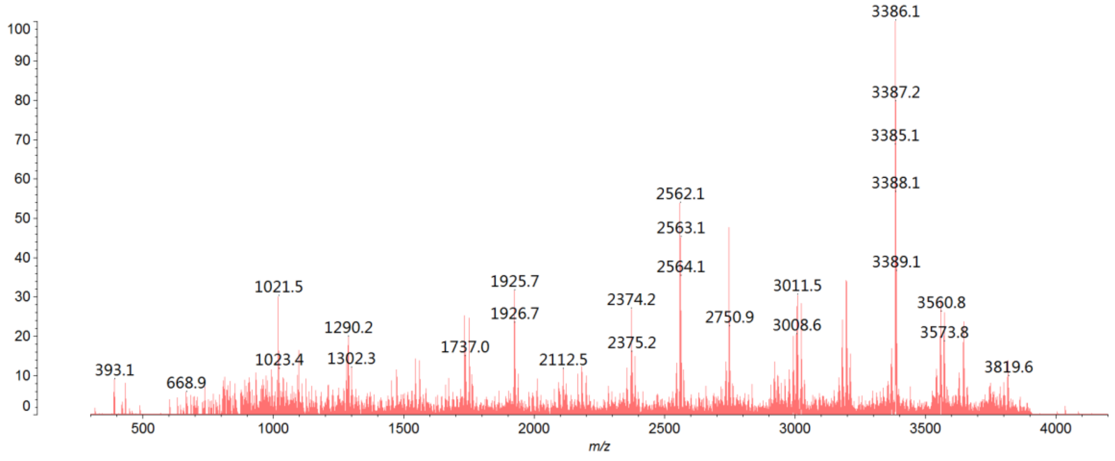

Figure S56, MALDI–TOF/TOF fragment ion spectra of the parent ions at m /z 4027

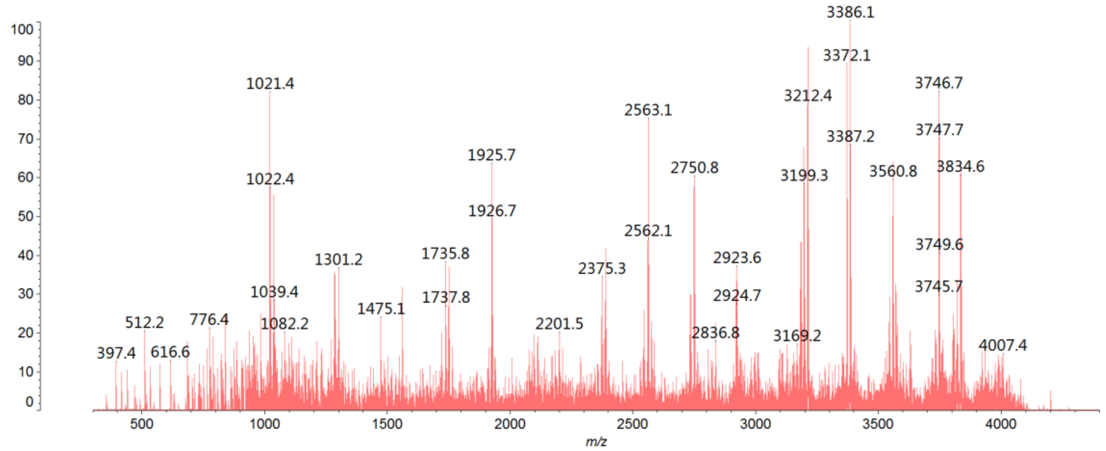

Figure S57, MALDI–TOF/TOF fragment ion spectra of the parent ions at m /z 4213

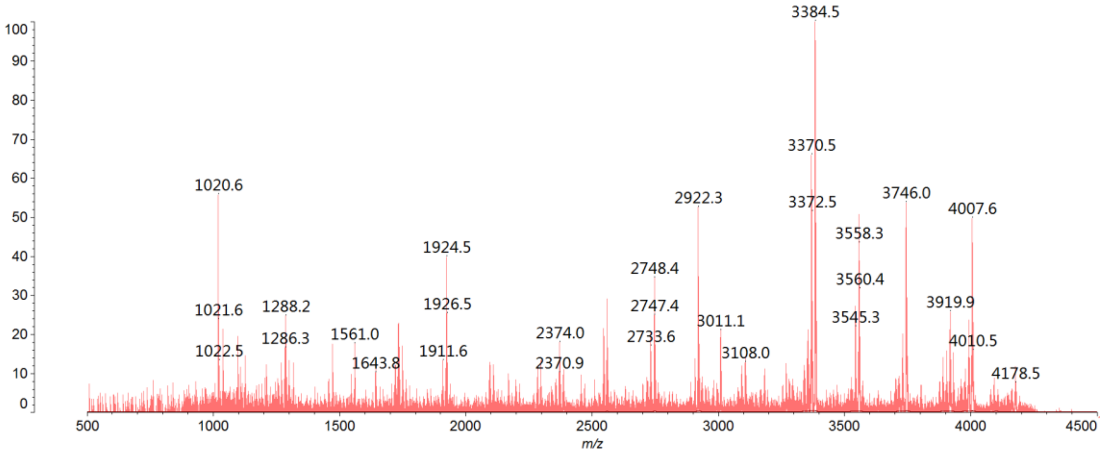

Figure S58, MALDI–TOF/TOF fragment ion spectra of the parent ions at m /z 4387

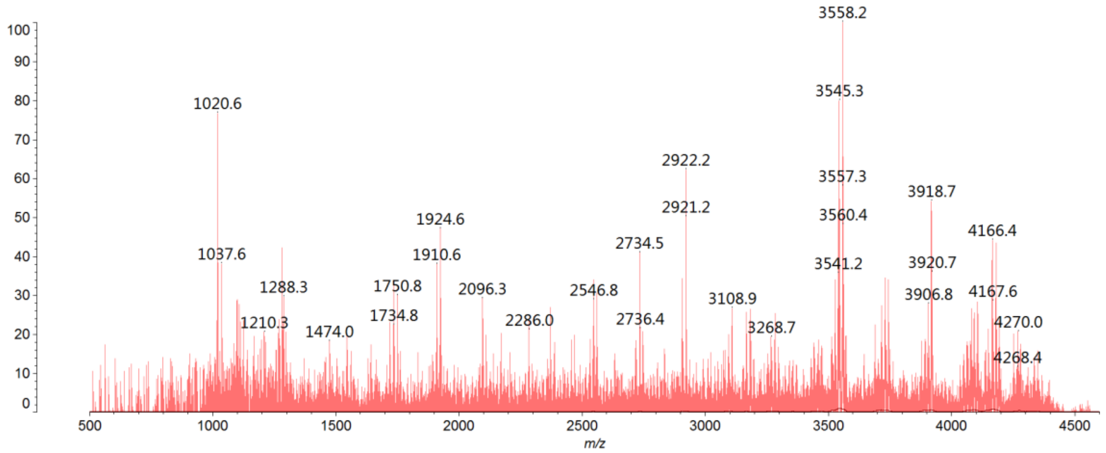

Figure S59, MALDI–TOF/TOF fragment ion spectra of the parent ions at m /z 4561

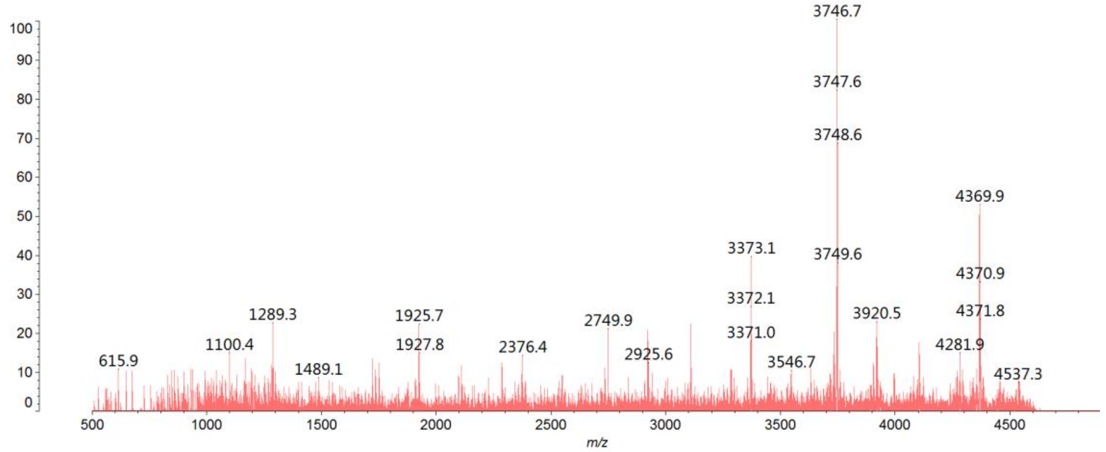

Figure S60, MALDI–TOF/TOF fragment ion spectra of the parent ions at m /z 4748

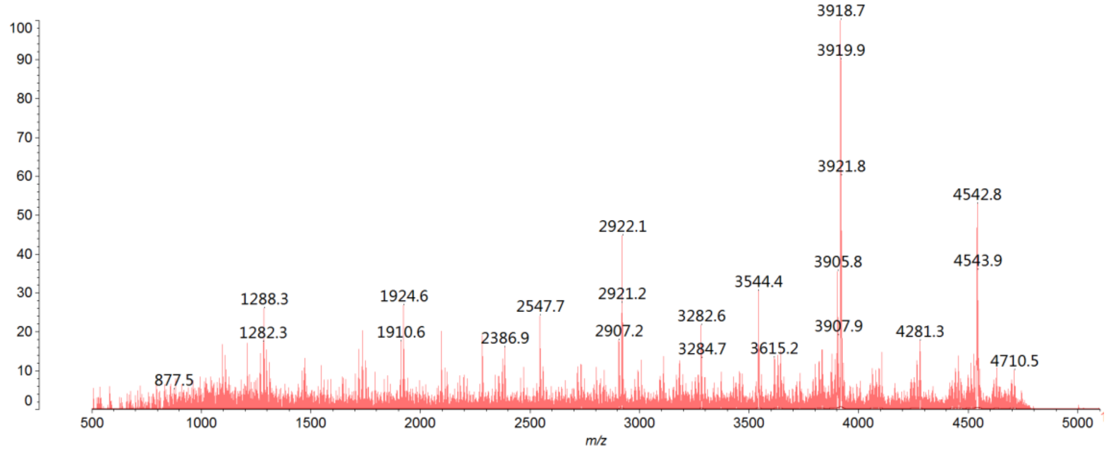

Figure S61, MALDI–TOF/TOF fragment ion spectra of the parent ions at m /z 4922
